# Esrrb Is a Direct Nanog Target Gene that Can Substitute for Nanog Function in Pluripotent Cells

**DOI:** 10.1016/j.stem.2012.08.002

**Published:** 2012-10-05

**Authors:** Nicola Festuccia, Rodrigo Osorno, Florian Halbritter, Violetta Karwacki-Neisius, Pablo Navarro, Douglas Colby, Frederick Wong, Adam Yates, Simon R. Tomlinson, Ian Chambers

**Affiliations:** 1 MRC Centre for Regenerative Medicine, Institute for Stem Cell Research, School of Biological Sciences, University of Edinburgh, 5 Little France Drive, Edinburgh EH16 4UU, Scotland

## Abstract

Embryonic stem cell (ESC) self-renewal efficiency is determined by the level of Nanog expression. However, the mechanisms by which Nanog functions remain unclear, and in particular, direct Nanog target genes are uncharacterized. Here we investigate ESCs expressing different Nanog levels and *Nanog*^*−/−*^ cells with distinct functionally inducible Nanog proteins to identify Nanog-responsive genes. Surprisingly, these constitute a minor fraction of genes that Nanog binds. Prominent among Nanog-reponsive genes is Estrogen-related receptor b (Esrrb). Nanog binds directly to *Esrrb,* enhances binding of RNAPolII, and stimulates *Esrrb* transcription. Overexpression of Esrrb in ESCs maintains cytokine-independent self-renewal and pluripotency. Remarkably, this activity is retained in *Nanog*^−/−^ ESCs. Moreover, Esrrb can reprogram *Nanog*^−/−^ EpiSCs and can rescue stalled reprogramming in *Nanog*^−/−^ pre-iPSCs. Finally, Esrrb deletion abolishes the defining ability of Nanog to confer LIF-independent ESC self-renewal. These findings are consistent with the functional placement of Esrrb downstream of Nanog.

## Graphical abstract

**Graphical Abstract:**

**Figure:**
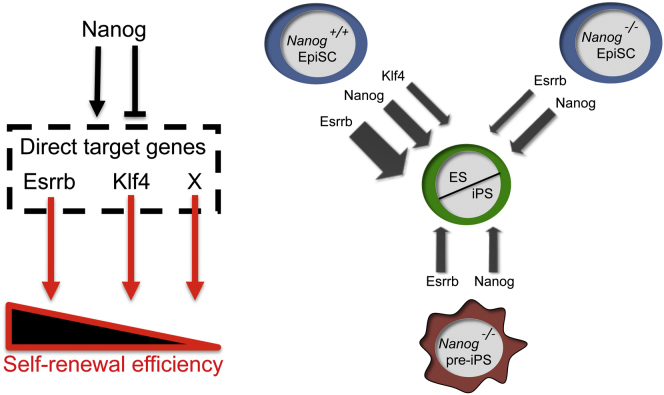

**Highlights:**

► Direct transcriptional targets of Nanog are identified, including, prominently, Esrrb ► Esrrb can maintain pluripotency independently of cytokine or Nanog ► Esrrb can reprogram Nanog null cells to naive pluripotency ► Esrrb is required for Nanog to confer cytokine independence on ESCs

## Teaser

The transcription factor Esrrb is a major functional downstream target of Nanog in the pluripotency network, and can replace Nanog in both the maintenance of pluripotency and reprogramming.

## Introduction

Self-renewal of mouse embryonic stem cells (ESCs) is under the intrinsic control of a gene regulatory network centered on the transcription factors Oct4, Sox2, and Nanog (Chen et al., 2008; Kim et al., 2008; Loh et al., 2006) (reviewed in Chambers and Tomlinson, 2009; Jaenisch and Young, 2008). Since its identification (Chambers et al., 2003; Mitsui et al., 2003), Nanog has been considered a central player in the specification of pluripotent cell identity in vivo (Mitsui et al., 2003; Silva et al., 2009) and in the control of efficient self-renewal of pluripotent cells in vitro (Boyer et al., 2005; Chambers et al., 2003, 2007; Ivanova et al., 2006; Loh et al., 2006). As well as the defining functional feature of conferring cytokine-independent self-renewal when overexpressed (Chambers et al., 2003), Nanog is able to increase reprogramming efficiency in cell hybrid experiments (Silva et al., 2006) and is required for somatic cells to be reprogrammed to naive pluripotency (Silva et al., 2009).

Individual ESCs fluctuate between states of high Nanog expression, associated with high self-renewal efficiency, and low Nanog expression, associated with an increased propensity to differentiate (Chambers et al., 2007). These functional differences are likely to be determined by the differential expression of Nanog target genes. Therefore, it is of particular importance to identify such target genes and to determine their biological contribution to Nanog function. With this aim we used complementary transcriptional profiling strategies to identify potential targets of Nanog. One of the most prominent genes identified in this analysis is Esrrb, an orphan nuclear receptor that is part of the pluripotency gene regulatory network (Chen et al., 2008; Ivanova et al., 2006; Kim et al., 2008; Loh et al., 2006; van den Berg et al., 2008, 2010; Wang et al., 2006; Zhang et al., 2008). Esrrb is involved in ESC self-renewal (Ivanova et al., 2006; Loh et al., 2006) and has been shown to promote reprogramming of mouse embryonic fibroblasts (Feng et al., 2009). However, the regulation of *Esrrb* in ESCs and the details of Esrrb function in sustaining pluripotency and promoting reprogramming are not well understood. We therefore investigated the regulation of *Esrrb* and the role of Esrrb in ESC self-renewal and cellular reprogramming using wild-type, Nanog mutant, and *Esrrb* mutant cells. Our results highlight the important functional interactions between Esrrb and its upstream regulator Nanog in the context of ESC self-renewal and pluripotency.

## Results

### The Transcriptional Network Downstream of Nanog

**Figure 1:**
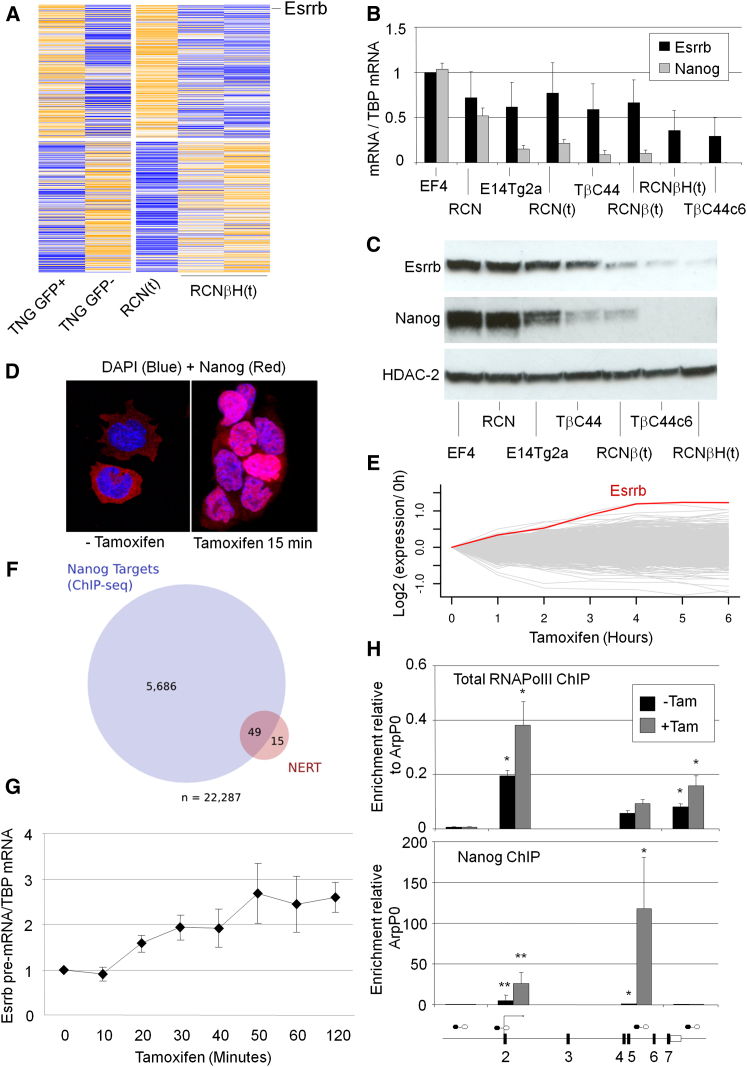
Identification of Nanog Target Genes Including Esrrb (A) Deep-SAGE profile of sorted Nanog-positive (GFP+) and Nanog-negative (GFP–) TNG cells, ESCs with wild-type levels of Nanog expression (RCN(t)) and *Nanog*^−/−^ ESCs (RCNβH(t)). Genes were ranked according to the expression level and fold difference in expression in TNG+ versus TNG− and RCN(t) versus RCNβH(t); the plot shows the first 250 most upregulated (top) or downregulated (bottom) genes. Colors: yellow, expression above average; blue, below average. (B) Esrrb transcript levels in two cell lines overexpressing Nanog (EF4 and RCN), two cell lines with wild-type *Nanog* (E14Tg2a and RCN(t)), two *Nanog*^+/−^ cell lines (TβC44 and RCNβ(t)), and two *Nanog*^−/−^ cell lines (TβC44Cre6 and RCNβH(t)). Error bars: standard deviation (n = 4). (C) Immunoblot analysis of Esrrb and Nanog levels in the same ESC lines. (D) Immunohistochemical analysis of the intracellular localization of Nanog in ESΔN-NERT cells in response to 1 μM tamoxifen as indicated. (E) Global transcriptional changes after ESΔN-NERT stimulation with tamoxifen as indicated; the Esrrb changes are in red. Mean expression levels in three independent experiments are shown. (F) Venn diagram showing the intersection of significantly upregulated or downregulated genes identified in (E) compared to genes bound by Nanog according to two independent genome-wide ChIP studies. (G) Esrrb pre-mRNA kinetics in ESΔN-NERT cells stimulated with tamoxifen as indicated. Error bars: standard deviation of expression values in three different clones. (H) Chromatin from ESΔN-NERT cells treated with 1 μM tamoxifen for 0 or 24 hr was immunoprecipitated with Nanog or total RNAPolII antibodies. Enrichment relative to the ArpP0 promoter is measured using the primers indicated at *Esrrb*. Error bars: standard deviation (n = 3); ^∗^p ≤ 0.05, ^∗∗^p ≤ 0.01. See also Figures S1 and S2 and Tables S1.1 and 1.2.

To identify genes controlled by Nanog, we compared the transcriptional profiles of ESCs in which GFP has been knocked in to one of the *Nanog* alleles (TNG cells; Chambers et al., 2007) that were sorted into SSEA1^+^/GFP^high^ and SSEA1^+^/GFP^low^ populations, together with *Nanog*^*+/+*^ and *Nanog*^*−/−*^ cells (Chambers et al., 2007) (Figure 1A). Good agreement between duplicate samples of *Nanog*^*−/−*^ RNA indicated reliable output from the Deep-SAGE protocols. Moreover, broad agreement was observed between both *Nanog*^−/−^ and Nanog:GFP^−^ as well as between *Nanog*^+/+^ and Nanog:GFP^+^ cells. Of 500 genes showing the greatest change in expression, *Esrrb* was the transcription factor that showed the closest positive correlations with Nanog and consistent variations in both Nanog:GFP^+^ versus Nanog:GFP^−^ and wild-type versus *Nanog*^−/−^ comparisons (fold change ≥1.5), closely followed by Klf4 (Table S1.1). To better understand the role of Esrrb in ESC pluripotency, we further characterized the expression of the *Esrrb* gene in ESCs and its regulation by Nanog.

The mouse *Esrrb* gene has six coding exons, with evidence for four alternatively spliced Esrrb mRNAs in the ENSEMBL EST databases (Figure S1A available online). To determine which of these transcripts are expressed in ESCs, quantitative PCR (Q-PCR) was used to amplify junctions between the coding exons and the alternative 5′ and 3′ untranslated regions (UTRs) (Figure S1A). In ESCs, the most abundant transcript includes the 5′UTR adjacent to the coding portion of exon 2 and the 3′UTR in exon 7 (Figures S1A and S1B).

**Figure 2:**
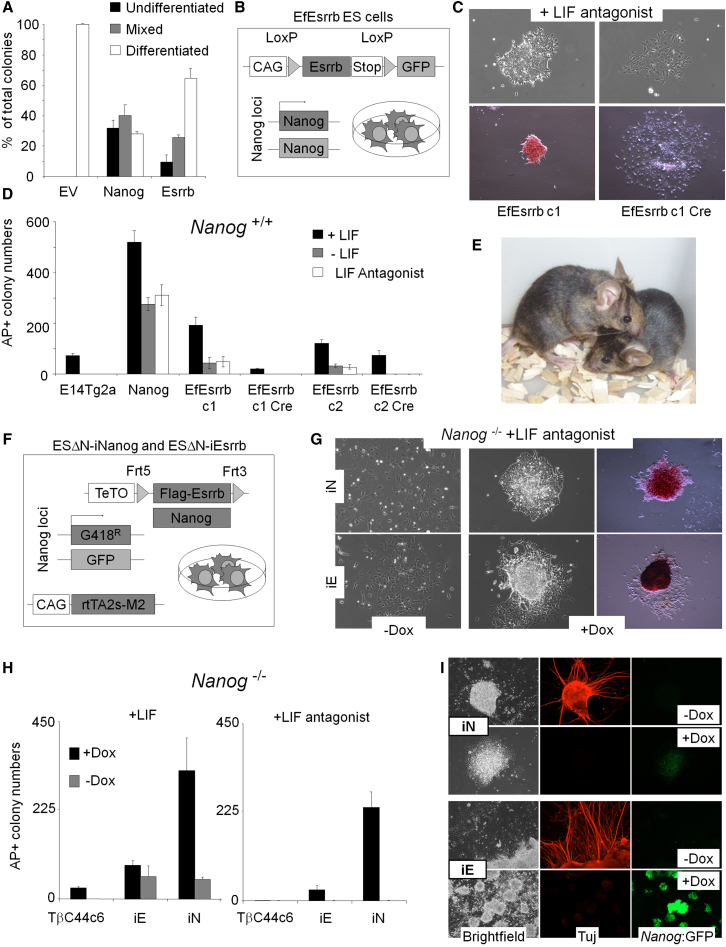
Esrrb Overexpression Confers LIF and Nanog-Independent Self-Renewal (A) *lifr*^−/−^:PyLT^+^ LRK1 cells were transfected with episomal plasmids encoding the indicated ORF (EV; empty vector) and the number of AP-positive colonies was determined after clonal density plating in the absence of IL-6/sIL6R. Error bars: standard deviation (n = 3). (B) Schematic representation of EfEsrrb ESCs. (C) Colony morphology (top) and AP staining (bottom) of EfEsrrb c1 cultured in the presence of hLIF-05. (D) E14Tg2a, Nanog-, and Esrrb-overexpressing cells before and after Cre reversion were plated at clonal density and cultured in the presence or absence of LIF or hLIF-05 for 7 days, and the number of AP-positive colonies was counted. Error bars: standard deviation (n = 3). (E) Chimeras generated after injection into C57BL/6 blastocysts of EfEsrrb-Cre ESCs passaged twice at clonal density in the presence of hLIF-05 and transfected with a Cre expression vector to excise the Esrrb transgene. (F) Schematic representation of the genetic manipulations used to make ESΔN-iNanog or ESΔN-iEsrrb cells. (G) Colony morphology of ESΔN-iNanog (iN) or ESΔN-iEsrrb (iE) cells plated at clonal density and cultured in the presence of hLIF-05 (+/− doxycycline) for 8 days. Right hand panels: AP staining of colonies formed in the presence of doxycycline. (H) Number of AP-positive colonies formed after clonal density plating of ESΔN-iNanog (iN) or ESΔN-iEsrrb (iE) cells in the presence of LIF or hLIF-05 and cultured (+/− doxycycline) for 8 days. Error bars: standard deviation (n = 3). (I) ESΔN-iNanog (iN) and ESΔN-iEsrrb (iE) cells in a neural differentiation protocol, without (top rows) or with (bottom rows) doxycycline for 9 days. Cells were fixed, stained for βIII-Tubulin (Tuj), and analyzed by fluorescence microscopy. See also Figures S3 and S4 and Table S2.

Different ESC lines in a Nanog mutant series (Chambers et al., 2003, 2007) showed a correlation between Nanog expression and levels of Esrrb mRNA (Figure 1B) and protein (Figure 1C). These variations in Esrrb mRNA levels reflect transcriptional control of *Esrrb* by Nanog rather than RNA stabilization, since differences in mRNA level (Figure S1C) were also seen for the pre-mRNA (Figure S1D). Furthermore, tamoxifen-induced elimination of Nanog from ESCs (Chambers et al., 2007) results in decreased Esrrb mRNA expression, an effect not attributable to differentiation as shown by stable Oct4 levels (Figure S1E). To investigate the dynamics of Nanog control of Esrrb transcription, we measured Esrrb mRNA levels in TβC44Cre6 *Nanog*^−/−^ ESCs expressing a tamoxifen-regulatable Nanog-ER^T2^ fusion protein (ESΔN-NERT, Figure S2A). In these cells Nanog nuclear relocalization is induced within 15 min of tamoxifen addition (Figure 1D). Three independent ESΔN-NERT lines induced Esrrb mRNA and protein at levels that correlated to the level of Nanog-ER^T2^ mRNA expression (Figures S1F and S1G). Tamoxifen treatment of ESΔN-NERT cells resulted in self-renewal in the absence of LIF to an extent comparable to that induced by wild-type Nanog expression (Figure S1I) in an identical *Nanog*^−/−^ background (Figures 2F and S2A), indicating that Nanog-ER^T2^ is fully functional.

To investigate the dynamics of Nanog control of transcription genome-wide, microarray analyses were performed at 1 hr time intervals over a 6 hr period following Nanog nuclear relocalization in ESΔN-NERT c3 cells. Sixty-four genes showed a differential gene expression pattern (≥1.5-fold change, p ≤ 0.05) during the time course (Figure 1E; Table S1.2). This is of interest given that thousands of binding sites for Nanog have been identified in genome-wide ChIP studies (Chen et al., 2008; Kim et al., 2008; Marson et al., 2008). We therefore compared the overlap between the Nanog-sensitive genes identified in our analysis with the common Nanog-bound targets identified in ChIP-Seq studies using our recently generated, publically available GeneProf software (Halbritter et al., 2012). The vast majority of the Nanog-sensitive genes that we identified were present in both ChIP-Seq studies (Chen et al., 2008; Marson et al., 2008), but 99% of the genes identified as putative Nanog targets by ChIP are insensitive to changes in Nanog over the time course of our study (Figure 1F).

Microarray analyses were also performed following induction of wild-type Nanog in *Nanog*^−/−^ ESΔN-iNanog ESCs (which carry a doxycycline-inducible Nanog transgene; Figure 2F). Since full transcript induction in ESΔN-iNanog cells is achieved by 6 hr (Figure S2B), microarray analysis used cells induced for 0, 6, or 12 hr. In this system, only 31 genes showed ≥1.5-fold change in expression after 12 hr of induction (p ≤ 0.05) (Figure S2C and Table S1.3). The lower number of identified genes is likely to result from the slower induction of nuclear Nanog in ESΔN-iNanog compared to ESΔN-NERT cells. The vast majority of targets (21/31) were also identified in ESΔN-NERT cells and 8/10 of the remaining genes are also differentially expressed in ESΔN-NERT cells but with <1.5-fold change. Together these analyses identify a reliable list of Nanog-responsive genes with which to explore the mechanisms of Nanog activity in ESCs.

Strikingly, Esrrb is the transcript showing the most pronounced induction in the ESΔN-NERT microarray (Figure 1E; Table S1.2) and the strongest induced transcription factor in ESΔN-iNanog cells (Figure S2E; Table S1.3). Of the other 63 targets identified in ESΔN-NERT cells, 10 are transcription factors expressed at significant levels. Of these, the closest transcription factor to change after Esrrb in both ESΔN-NERT and ESΔN-iNanog cells is Klf4, the only other transcription factor to show a consistent positive change (≥1.5-fold) in all other data sets (Table S1).

Q-PCR confirmed the rapid induction of Esrrb mRNA by Nanog-ER^T2^ (Figure S1H) and detected increased Esrrb pre-mRNA within 20 min of tamoxifen treatment (Figure 1G), arguing in favor of a direct role for Nanog in *Esrrb* transcription. Moreover, tamoxifen treatment of ESΔN-NERT cells not only stimulated binding of Nanog-ER^T2^ to *Esrrb* (Figure 1H) but also resulted in a 2-fold increase in RNAPolII recruitment to the *Esrrb* promoter (Figure 1H). These results establish *Esrrb* as a major positive target of direct transcriptional activation by Nanog in ESCs.

### Esrrb Overexpression Confers Cytokine-Independent Self-Renewal in the Absence of Nanog

The observation that Nanog lies upstream of Esrrb prompted us to investigate whether the cytokine independence conferred upon ESCs by Nanog overexpression (Chambers et al., 2003) might be mediated by Esrrb. Supertransfection of *lifr*^−/−^ cells (Chambers et al., 2003) with an episomal Esrrb expression vector resulted in self-renewal in the absence of IL6/sIL6R (Figure 2A). Integration of a loxP-flanked Esrrb transgene (Figure 2B) allowed the isolation of cell lines that overexpress Esrrb reversibly (EfEsrrb cells) (Figure S3A). These cells showed a constitutive capacity to form undifferentiated alkaline phosphatase (AP)-positive self-renewing colonies in the presence of the LIF antagonist hLIF-05 (Vernallis et al., 1997) (Figures 2C and 2D), a phenotype reversed by Cre expression (Figures 2C and 2D). To rigorously determine whether Esrrb overexpression is sufficient to maintain pluripotency through clonal expansion in the absence of LIF signaling, EfEsrrb cells were plated at clonal density in the presence of LIF antagonist and passaged twice at clonal density. At this point, control parental cells had completely differentiated and could not be passaged further. In contrast, EfEsrrb clones continued to self-renew. These cells were treated with Cre, and GFP-expressing cells that had deleted the Esrrb ORF (Figure 2B) were expanded in LIF. Injection of these cells into C57BL/6 blastocysts gave rise to adult chimeras (Figure 2E). Therefore, Esrrb is able to functionally substitute for Nanog overexpression to sustain gp130-independent self-renewal.

A more precise comparison of self-renewal induced by overexpression of Nanog, Esrrb, and Klf4 (the second transcription factor showing closest correlation with Nanog in our analysis) was obtained using recombinase-mediated cassette exchange (RMCE) to introduce doxycycline-inducible transgenes into the same locus of E14Tg2a cells (Figure S3B; details in Experimental Procedures). Cells were plated at clonal density with or without LIF, in increasing doxycycline concentrations. Maximal self-renewal efficiency was observed at 3 μg/ml doxycycline for Nanog and Klf4, but at 1 μg/ml for Esrrb, with excessive Esrrb expression stimulating differentiation (Figures S3C and S3D). These results indicate that the self-renewal phenotypes directed by overexpression of Esrrb and Nanog were comparable with both surpassing Klf4.

The ability of Esrrb to direct cytokine-free self-renewal independent of Nanog expression was next tested. Clonal derivatives of the *Nanog*^−/−^ line TβC44Cre6 were obtained that had integrated a constitutively expressed Esrrb transgene (Figures S3E and S3F). These cell lines form undifferentiated colonies when plated without LIF at clonal density (Figures S3G and S3H). Addition of LIF to Esrrb-overexpressing cells increased clonal self-renewal efficiency. Therefore, Esrrb acts cooperatively with LIF but can act independently of Nanog.

To more precisely compare self-renewal induced by Esrrb or Nanog overexpression in *Nanog*^−/−^ cells, RCME was used to introduce doxycycline-inducible Nanog or Esrrb transgenes into the same locus in TβC44Cre6 cells (ESΔN-iNanog and ESΔN-iEsrrb cells; Figure 2F). This resulted in comparable levels of Nanog and Esrrb mRNAs following doxycycline treatment (Figure S4A). These cells were plated at clonal density in ESC medium supplemented with LIF or LIF antagonist, in the presence or absence of doxycycline. Induction of Esrrb or Nanog resulted in the formation of undifferentiated AP-positive colonies in the complete absence of LIF signaling (Figure 2G). Interestingly, a 5-fold greater self-renewal efficiency was seen when Nanog rather than Esrrb was induced from the same locus (Figure 2H). These results show that while Esrrb can act independently of Nanog, restoring Nanog expression in *Nanog*^−/−^ ESCs has a greater effect on self-renewal efficiency.

Nanog overexpression affects the ability of ESCs to differentiate in vitro (Chambers et al., 2003). To determine whether Esrrb overexpression has a similar phenotype, ESΔN-iNanog and ESΔN-iEsrrb lines were cultured in N2B27. Overt neural differentiation was observed for ESΔN-iNanog and ESΔN-iEsrrb cells in the absence of transgene induction. In contrast, doxycycline treatment of ESΔN-iNanog or ESΔN-iEsrrb cultures blocked neural differentiation (Figure 2I).

Doxycycline-treated ESΔN-iNanog and ESΔN-iEsrrb cells could be passaged in the presence of LIF antagonist for more than 1 month (Figure S4B) and retained the ability to form teratocarcinomas composed of representative tissues of all three primary germ layers as well as undifferentiated embryonal carcinoma (EC) upon transplantation to mice (Figure S4C; Table S2). Therefore, Esrrb is able to maintain ESC pluripotency through multiple passages without gp130 signaling and even in the absence of Nanog.

### Esrrb Reverts EpiSCs to Chimera-Competent Pluripotency

**Figure 3:**
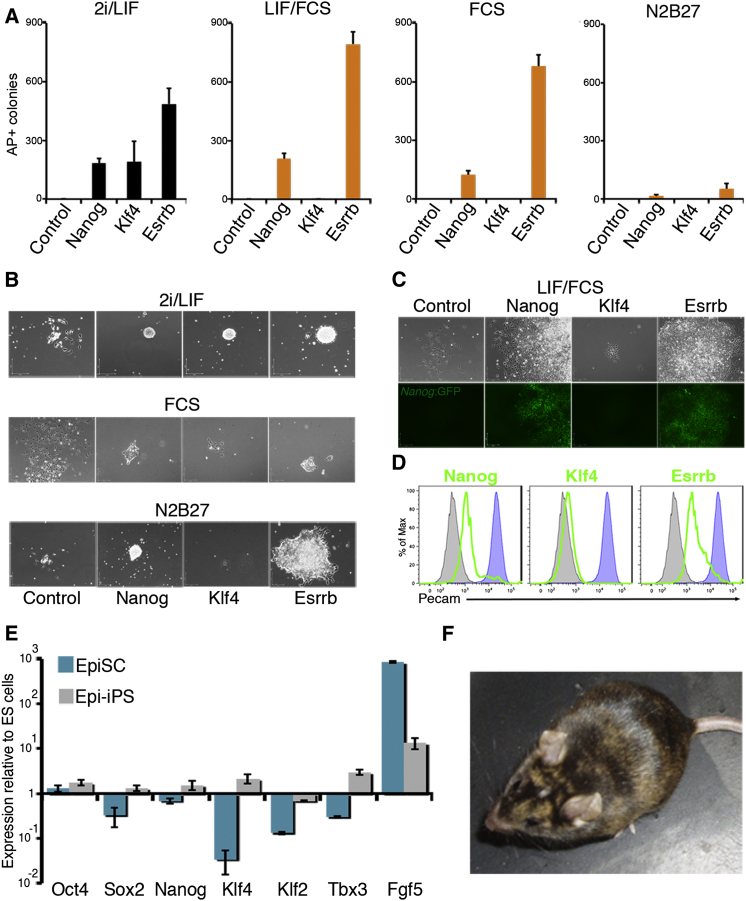
Expression of Esrrb Reverts EpiSCs to Chimera Competency (A) AP-positive colony formation by Epi-iPSCs. EpiSCs expressing polyoma large T-antigen were transfected with episomal vectors encoding empty vector (EV), Nanog, Klf4, or Esrrb, plated in the indicated medium containing puromycin, and stained for AP after 7 days. Error bars: standard deviation (n = 3). (B) Morphology of primary Epi-iPSC colonies formed after transfection of the respective episomal vector and culture in the indicated medium for 7 days. (C) Morphology and *Nanog*:GFP expression of primary Epi-iPSC colonies formed after transfection of the respective episomal vector and culture in FCS/LIF/GMEMβ for 7 days. (D) FACS analysis of Pecam1 expression 7 days after transfection of the indicated DNAs. TNG/T ESCs (blue) and EpiSCs (gray) were used as controls for Pecam1 expression. (E) mRNA expression in E14/T EpiSC and Epi-iPSC colonies expanded in the absence of selection after episomal expression of Esrrb and medium switch into FCS/LIF/GMEMβ. Error bars: standard deviation of gene expression in three independent experiments. (F) Chimeric mouse obtained from blastocyst injection of Esrrb-induced Epi-iPSCs. See also Figure S5 and Table S3.

It has been shown that Nanog or Klf4 overexpression can reprogram EpiSCs to ESC pluripotency (Guo et al., 2009; Silva et al., 2009). Therefore, the abilities of Nanog, Esrrb, and Klf4 to mediate the reversion of EpiSCs to an ESC state were compared. Episomal expression of Nanog, Esrrb, or Klf4, coupled with removal of Activin/Fgf, could induce reversion of EpiSCs to an ESC-like state (Figure 3A). Esrrb displayed a higher reprogramming efficiency than Nanog or Klf4 (Figure 3A). Furthermore, Nanog and Esrrb allowed AP-positive colony formation in all conditions (Figure 3A), whereas Klf4 could only revert EpiSCs to ESC pluripotency when combined with LIF/2i (Figure 3A). Primary Epi-iPSC colonies displayed an undifferentiated morphology (Figure 3B) and in FCS/LIF/GMEMβ, Nanog and Esrrb, but not Klf4, induced the re-expression of *Nanog*:GFP (Figure 3C) and Pecam1 (Figure 3D), a cell surface marker expressed in the inner cell mass (ICM)/ESCs and downregulated in the epiblast/EpiSCs (Hayashi et al., 2008; Robson et al., 2001). To further characterize the Esrrb-induced Epi-iPSCs, clones were picked and expanded in FCS/LIF/GMEMβ. Expression of Nanog, Sox2, Klf4, Klf2, and Tbx3 were restored to ESC levels, while expression of the early marker of differention Fgf5 was reduced (Figure 3E). Injections of the Esrrb-reverted Epi-iPSCs into blastocysts produced adult chimeras, indicating that enforced Esrrb expression can restore chimera-forming potential to EpiSCs (Figure 3F; Table S3).

To investigate the reproducibility of these findings, plasmids containing loxP-flanked Nanog, Esrrb, or Klf4 ORFs upstream of GFP (Figure S5A) were integrated into RC EpiSCs that constitutively express tamoxifen-inducible Cre recombinase (Cre-ER^T2^) from *ROSA26* (RC = RosaCre). Overexpression of Nanog, Esrrb, or Klf4 was verified by Q-PCR (Figure S5B). Populations were then switched to 2i/LIF/N2B27. ESC-like colonies were obtained, with Esrrb displaying a 5-fold higher reprogramming efficiency than Nanog or Klf4 (Figure S5C). Esrrb-induced Epi-iPSC clones were treated with tamoxifen and transgene deletion was monitored by GFP expression (Figure S5D). Pecam1 re-expression in Esrrb-induced Epi-iPSCs was maintained following transgene excision, suggesting stable reprogramming to an ESC state (Figure S5E). Following Cre excision of Esrrb, cells became dependent on LIF for colony formation and displayed heterogenous expression of Nanog, Esrrb, and Klf4 (Figures S5F and S5G). These results show that Esrrb expression reinstates ESC pluripotency in EpiSCs.

### Esrrb Can Reprogram *Nanog*^−/−^ EpiSCs to Chimera Competency

**Figure 4:**
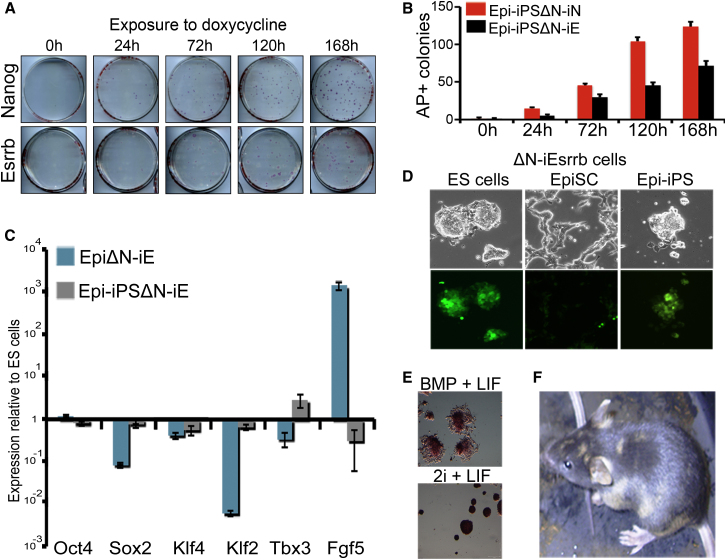
Nanog Null EpiSC Are Reverted to Naive Pluripotency by Esrrb Expression (A) *Nanog*^−/−^ EpiSCs carrying doxycycline-inducible Nanog or Esrrb transgenes were plated in FCS/LIF/GMEMβ with doxycycline for the indicated times. After 7 days, plates were stained for AP. (B) Scoring of the AP colonies obtained from the experiment described in (A). Error bars: standard deviation (n = 3). (C) mRNA expression in uninduced EpiΔN-iEsrrb and the reverted Epi-iPSΔN-iEsrrb ESC-like colonies obtained by induction of Esrrb and expansion in the absence of selection and doxycycline. Error bars: standard deviation of gene expression in two independent experiments. (D) Brightfield (top panels) and fluorescence (bottom panels) images of ESΔN-iEsrrb, EpiΔN-iEsrrb, and Epi-iPSΔN-iEsrrb cells. (E) AP-positive colonies of Epi-iPSΔN-iEsrrb cells grown in N2B27 supplemented with BMP/LIF (top) or 2i/LIF (bottom). (F) Chimeric mouse obtained from a blastocyst injection with Epi-iPSΔN-iEsrrb cells. See also Table S3.

Nanog is dispensable for the establishment and maintenance of primed pluripotency (Osorno et al., 2012) but is required for the acquisition of naive pluripotency, since somatic *Nanog*^−/−^ cells cannot be converted into fully reprogrammed iPSCs (Silva et al., 2009). To determine whether Esrrb could revert EpiSCs to an ESC state in the absence of Nanog, ESΔN-iNanog and ESΔN-iEsrrb ESCs were converted into EpiSC lines (EpiΔN-iNanog and EpiΔN-iEsrrb) by passaging in Activin/FGF (Guo et al., 2009). This allowed comparative investigation of the abilities of Nanog and Esrrb to impose an ESC identity by simply applying doxycycline and removing Activin/Fgf. AP-positive Epi-iPSC colonies were obtained following induction of Nanog and, to our surprise, also following Esrrb induction (Figure 4A). However, whereas Esrrb induced EpiSC reprogramming with greater efficiency than Nanog in wild-type cells, the opposite was observed in *Nanog*^−/−^ cells (Figures 4A and 4B), suggesting that Nanog is required for maximal Esrrb efficacy. AP-positive colonies were obtained after as little as 24 hr exposure to doxycycline of both EpiΔN-iNanog and ESΔN-iEsrrb cells with a clear correlation between the doxycycline treatment period and the number of Epi-iPSC colonies obtained (Figures 4A and 4B). Esrrb-induced Epi-iPSΔN-iEsrrb clones were picked and expanded in the absence of further transgene induction and had reacquired expression of Sox2, Klf2, and Tbx3 and downregulated Fgf5 (Figure 4C). Importantly, Epi-iPSΔN-iEsrrb cells reacquired both ESC morphology and levels of *Nanog*:GFP similar to those in ESCs (Figure 4D). Epi-iPSΔN-iEsrrb cells could also form self-renewing AP-positive colonies in BMP/LIF and 2i/LIF (Figure 4E). Consistent with these findings, Epi-iPSΔN-iEsrrb cells injected into blastocysts produced adult chimeras (Figure 4F; Table S3). These results demonstrate that Esrrb can functionally substitute for the hitherto unique capacity of Nanog to reprogram *Nanog*^*−/−*^ cells to naive pluripotency.

### Esrrb Can Reprogram *Nanog*^−/−^ Neural Stem Cells

Reprogramming of neural stem cells (NSCs) has previously been reported to depend on Nanog (Silva et al., 2009). To ascertain if Esrrb overexpression could also promote reprogramming of NSCs, the efficiency of formation of hybrid colonies capable of being propagated in ESC medium (Silva et al., 2006) was compared following fusion of E14/T NSCs with wild-type ESCs or ESCs overexpressing Esrrb (Figure 2B) or Nanog. Overexpression of Esrrb stimulated formation of pluripotent hybrid colonies with a similar efficiency as that observed with Nanog overexpression (Figures S6A and S6B).

To determine whether the reprogramming capacity of Esrrb required the presence of Nanog in either fusion partner, we developed an experimental system in which *Nanog*^−/−^ NSCs are fused to *Nanog*^−/−^ ESCs overexpressing Esrrb. NSCs derived from *Nanog*^−/−^ RCNβH(t) can be propagated in NSC medium containing FGF/EGF (Conti et al., 2005) and show the characteristic vimentin-positive NSC morphology (Figure S6C). These *Nanog*^−/−^ NSCs were fused to ESΔN-CAGE (Figure S6D) and plated in ESC medium in the presence of puromycin and hygromycin to select for hybrids that reactivated *Nanog* transcription from the NSC genome. Control cell fusions of RCNβH(t) NSCs to Nanog and Esrrb overexpressing *Nanog*^+/+^ ESCs gave rise to 100–500 morphologically undifferentiated hybrid colonies per 10^6^ cells fused (Figure S6E; Table S4). However, no undifferentiated colonies were observed after fusion of RCNβH(t) NSCs with TβC44Cre6 ESCs. In contrast, fusions between ESΔN-CAGE ESCs and RCNβH(t) NSCs produced undifferentiated hybrid colonies that could be maintained in standard ESC medium through multiple passages (Figure S6E; Table S4).

**Figure 5:**
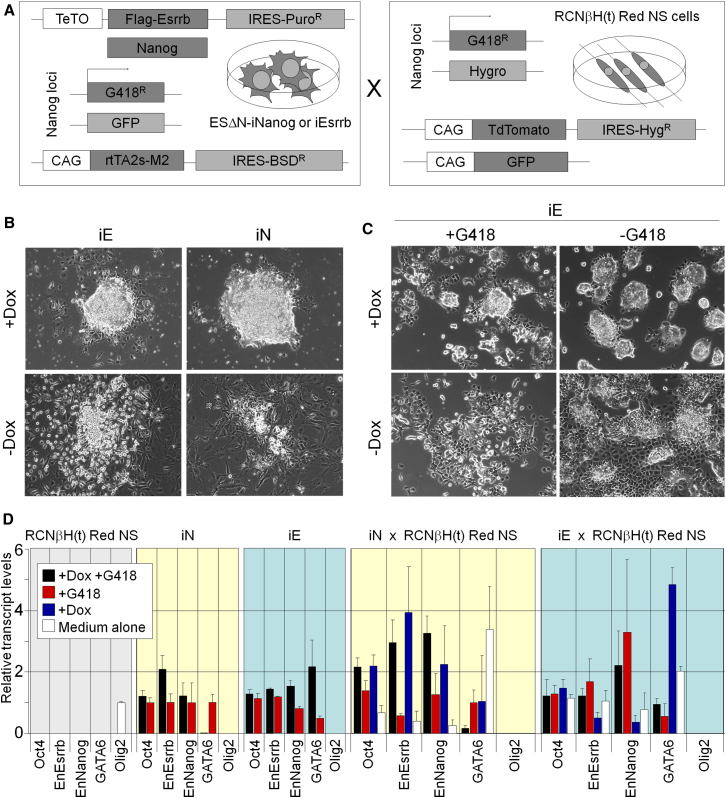
In Vitro Reprogramming by Cell Fusion Can Proceed in the Absence of Nanog (A) Schematic representation of the genetic manipulations performed on the lines used in the fusion experiments: ESΔN-iNanog and ESΔN-Esrrb cells and RCNβH(t) Red NSCs. (B) Colonies formed by ESΔN-iNanog (iN) or ESΔN-iEsrrb (iE) × RCNβH(t) Red NSCs hybrids after 16 days selection in blasticidin/hygromycin in the presence or absence of doxycycline. (C) Morphology of ESΔN-iEsrrb (iE) × RCNβH(t) Red NSC hybrids cultured in doxycycline or released from doxycycline for three passages (10 days) in the presence or absence of G418 to select for active *Nanog* transcription. (D) Gene expression profiles of endogenous genes in RCNβH(t) Red NSCs, ESΔN-iNanog (iN) cells or ESΔN-iEsrrb (iE) cells, and hybrid lines after three passages in the indicated conditions. Primers do not detect transgenes. Nanog primers bind to intron I, which is not deleted in the targeted alleles. Transcript levels are normalized to TBP and relative to expression in RCNβH(t) Red NS (Olig2) or ESΔN-iNanog cells cultured in G418 (all other genes). Error bars: ESC × NSC hybrids: standard deviation of gene expression in three independent clones. ESC and NSC lines: standard deviation of gene expression in two independent experiments. See also Figure S6 and Tables S4, S5, and S6.

To examine whether stable reprogramming of the NSC genome could be achieved without continued transgene expression, fusion experiments were performed using ESΔN-iNanog and ESΔN-iEsrrb cells. *Nanog*^−/−^ RCNβH(t) NSCs were transfected with a CAG-driven TdTomato-IRES-hygromycin^R^ construct. RCNβH(t) Red NSCs were fused with ESΔN-iNanog or ESΔN-iEsrrb cells (Figure 5A) and primary hybrids were replated in blasticidin and hygromycin. In the absence of doxycycline, only a small number of hybrid colonies were obtained (Table S5); these were predominantly differentiated (Figure 5B) and could not be expanded. In contrast, Nanog and Esrrb induction resulted in the formation of self-renewing AP-positive colonies (Figures 5B and S6G). Nanog induction promoted reprogramming at high frequency (∼300 colonies/million NSCs fused) as previously reported (Silva et al., 2006, 2009). In contrast, Esrrb overexpression resulted in a 10-fold lower reprogramming efficiency (Table S5). These differences were not due to altered fusion efficiencies, since similar results were obtained after replating sorted primary hybrids (Figure S6F; Table S6). Nonetheless, all reprogrammed hybrid lines could be expanded and cultured over multiple passages. Cells were then maintained or released from doxycycline and passaged in the presence or absence of G418 (to select for transcription from *Nanog*; Figure 5A). Hybrid lines could be serially passaged without continued Esrrb or Nanog in G418 (Figure 5C). In the absence of G418 selection, hybrid cells could be propagated without continued Esrrb or Nanog induction but had an increased tendency to differentiate, similar to *Nanog*^−/−^ ESCs (Chambers et al., 2007). This propensity was eliminated upon induction of Esrrb, identifying a further common feature between Esrrb and Nanog.

The stability of reprogramming of RCNβH(t) NSCs was investigated by analyzing gene expression in hybrid lines cultured in the presence or absence of doxycycline or G418 (Figure 5D). NSC-specific genes were silenced during reprogramming and were not re-expressed after transgene repression, while endogenous pluripotency genes were expressed in all lines analyzed even after withdrawal of doxycycline. Release of ESΔN-iEsrrb × RCNβH(t) hybrid lines from doxycycline and G418 resulted in an increased tendency to differentiate into primitive endoderm, as judged by morphology and GATA6 expression (Figures 5C and 5D). Despite this, culture in 2i/LIF/N2B27, a condition permissive only for completely undifferentiated cells, resulted in colonies with an undifferentiated morphology that could be serially passaged (Figure S6H). These data show that NSCs can be reprogrammed to pluripotency in the absence of Nanog by overexpression of Esrrb and that Esrrb is required to stabilize the reprogrammed hybrids but is dispensable once pluripotency is attained.

### Esrrb Can Complete Reprogramming of *Nanog*^−/−^ Somatic Cells to Naive Pluripotency

**Figure 6:**
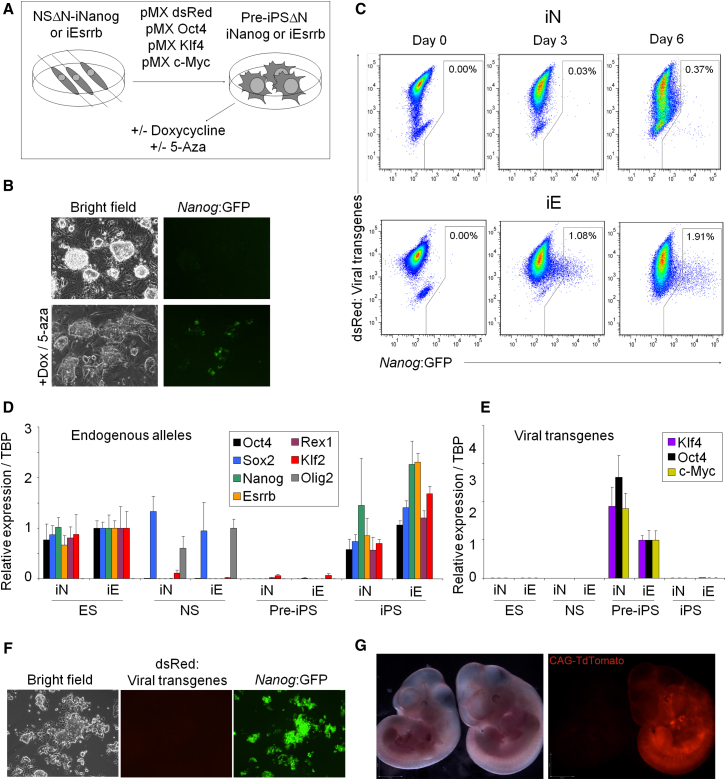
Esrrb Can Reprogram *Nanog*^−/−^ Somatic Cells to Naive Pluripotency (A) Experimental scheme used to derive pre-iPSCs and to induce completion of reprogramming. (B) Morphology and *Nanog*:GFP expression in pre-iPSΔN-iEsrrb cells cultured in the absence of doxycycline (top) or in doxycycline/5-azacytidine for 3 days (bottom). (C) FACS plots of viral transgene expression (dsRed) and *Nanog*:GFP in pre-iPSΔN-iNanog (iN) or pre-iPSΔN-iEsrrb (iE) cells treated with doxycycline/5-azacytidine as indicated. Percentages of cells positive for *Nanog*:GFP are shown. (D) Q-PCR of endogenous genes in ESΔN-iNanog (iN) or ESΔN-iEsrrb (iE) cells and derivative NSCs, pre-iPSCs, and iPSCs. Primers do not detect transgenes. Nanog primers bind to intron I, which remains in all targeted cells. All cell lines were maintained without doxycycline for at least three passages. mRNA levels (normalized to TBP) are relative to expression in NSΔN-iEsrrb cells (Olig2) or ESΔN-iEsrrb cells (all other genes). Error bars: iPSCs: standard deviation of gene expression in three independent clones. ESC, pre-iPSC, and NSC lines: standard deviation of gene expression in three independent experiments. (E) Q-PCR of retroviral transgenes in ESΔN-iNanog (iN) or ESΔN-iEsrrb (iE) cells and derivative NSCs, pre-iPSCs, and iPSCs. Primers do not detect endogenous transcripts. mRNA levels (normalized to TBP) are relative to expression in pre-iPSΔN-iEsrrb cells. Error bars: standard deviation of expression values in three independent experiments. (F) Morphology, dsRed, and *Nanog*:GFP expression in iPSΔN-iEsrrb cells cultured on gelatin without doxycycline for three passages. (G) Midgestation embryo obtained from blastocyst injection of iPSΔN-iEsrrb cells transfected with a ubiquitously expressed TdTomato transgene (right); control embryo (left). See also Table S7.

The ability of Esrrb to substitute for Nanog during transcription-factor-based induced pluripotency (Takahashi and Yamanaka, 2006) was next tested. Nanog is strictly required for completion of this process with *Nanog*^−/−^ cells stalling in an intermediate, pre-iPSC state in which they acquire the morphology and growth factor dependence of ESCs but do not express endogenous pluripotency genes or silence retroviral transgene expression (Silva et al., 2009). NSCs were generated from ESΔN-iNanog and ESΔN-iEsrrb ESCs and passaged ten times in NSC medium. These lines express the NSC marker Olig2 and Sox2 but not other pluripotency factors (Figure 6D). NSΔN-iNanog and NSΔN-iEsrrb cells were infected with retroviral vectors encoding Oct4, Klf4, c-Myc, and dsRed (to monitor LTR silencing upon completion of reprogramming; Figure 6A). Colonies resembling pre-iPSCs emerged at day 5 postinfection and could be maintained on feeders without reactivating *Nanog:*GFP (Figure 6B). Other pluripotency genes remained silenced and viral transgenes were expressed (Figures 6D and 6E). pre-iPSΔN-iNanog and pre-iPSΔN-iEsrrb cells were then treated with doxycycline to activate the Nanog or Esrrb transgenes. This was performed with or without 5-azacytidine, which has been shown to promote reprogramming (Huangfu et al., 2008) and facilitate the pre-iPSC to iPSC transition (Theunissen et al., 2011). Nanog induction in pre-iPSΔN-iNanog cells led to the emergence of *Nanog*:GFP^+^ cells by day 6 (Figure 6C). Strikingly, Esrrb induction resulted in faster, more pronounced reactivation of *Nanog:*GFP. For both pre-iPSΔN-iNanog and pre-iPSΔN-iEsrrb, G418-resistant, *Nanog*:GFP^+^ colonies could be picked and expanded without feeders or doxycycline. The resulting iPSΔN-iNanog and iPSΔN-iEsrrb lines resembled the parental ESC lines morphologically, were *Nanog:*GFP^+^/dsRed^–^ (Figure 6F), expressed endogenous pluripotency genes, and had silenced the viral transgenes (Figures 6D and 6E). Blastocyst injection of iPSΔN-iEsrrb cells resulted in contribution to midgestation embryos (Figure 6G; Table S7). These results demonstrate that Esrrb can drive completion of reprogramming in the absence of Nanog, indicating that Esrrb can substitute for *Nanog* in the acquisition of pluripotency.

### Esrrb and Nanog Share Target Genes

The results presented so far argue in favor of the existence of a degree of functional overlap between Esrrb and Nanog activity in pluripotent cells. Therefore, a comparison of the transcriptional programs activated in response to Nanog and Esrrb induction was performed by microarray analysis of doxycycline-treated ESΔN-iNanog and ESΔN-iEsrrb cells. An overall similar transcriptional response was detected upon Esrrb or Nanog elevation (Figure S2D) with 20% of the top 50 upregulated genes common between ESΔN-iNanog and ESΔN-iEsrrb cells (Figure S2E). The only transcription factor in this group was Klf4. Interestingly, Nanog induced Klf4 more effectively than Esrrb (Figure S2F). These results suggest that Esrrb can partially recapitulate Nanog activity by modulating a common set of transcriptional targets.

### Self-Renewal in Esrrb Knockout Cells

**Figure 7:**
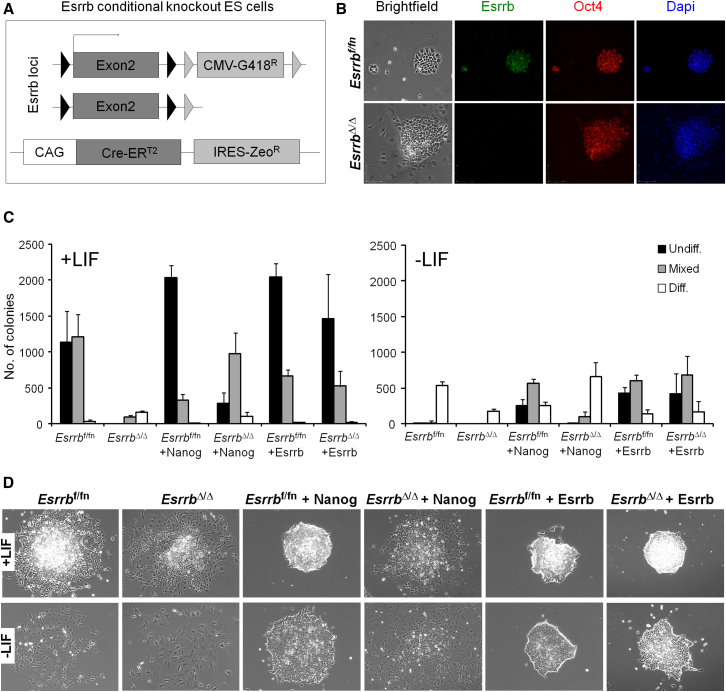
Loss of Esrrb Impairs Nanog-Driven LIF Independence (A) Schematic representation of the genetic manipulations used to make conditional knockout (*Esrrb*^f/fn^) ESCs that have two floxed *Esrrb* alleles and express Cre-ER^T2^. (B) Morphology and expression of Oct4 and Esrrb in *Esrrb*^f/fn^ and deleted *Esrrb*^Δ/Δ^ lines. (C) Colony formation after clonal density plating and 7 days culture (+/− LIF; values are the average of six independent clones for each indicated line). Error bars: standard deviation of the results obtained from six clones each analyzed in triplicate. (D) Representative morphologies of colonies formed by the indicated lines after 7 days of culture (+/− LIF). See also Figure S7.

To determine the requirement of Esrrb in ESC self-renewal, cells homozygous for a conditional Esrrb knockout allele (*Esrrb*^f/fn^) (Chen and Nathans, 2007) and expressing Cre-ER^T2^ were generated (Figures 7A, S7A, and S7B). Tamoxifen treatment of *Esrrb*^f/fn^ cells increases the degree of differentiation in these cultures. Nonetheless, stable *Esrrb*^Δ/Δ^ cell lines genetically devoid of Esrrb were readily isolated (Figure S7C and S7D). Although they show an impaired ability to self-renew in clonal assays (Figure 7C), *Esrrb*^Δ/Δ^ cells can be propagated in FCS/LIF/GMEMβ and maintain Oct4 expression (Figure 7B). These results establish the fact that despite having a clear stimulatory effect on the efficiency of colony formation, Esrrb is formally dispensable for ESC self-renewal.

Nanog was originally identified on the basis that overexpression conferred LIF-independent self-renewal (Chambers et al., 2003). The foregoing results indicate that Esrrb has a significant self-renewal function. If the hypothesis that Nanog mediates a significant portion of its self-renewal functions via Esrrb is correct, then Esrrb knockout should reduce the self-renewal efficiency conferred by Nanog overexpression. *Esrrb*^f/fn^ cells were transfected with a Nanog transgene or a control Esrrb transgene. Clonal overexpressing lines (*Esrrb*^f/fn^+Nanog and *Esrrb*^f/fn^+Esrrb) (Figures S7E and S7F) were treated with tamoxifen to induce deletion of *Esrrb* and stable knockout lines were derived (Figures S7C and S7D). Six Nanog and six Esrrb overexpressing *Esrrb*^f/fn^ and derivative *Esrrb*^Δ/Δ^ lines were tested for their ability to self-renew in the presence or absence of LIF in clonal assays (Figure 7C). Nanog overexpression resulted in the formation of undifferentiated colonies by *Esrrb*^f/fn^ ESCs in the absence of LIF. Enforced Esrrb expression gave comparable numbers of undifferentiated colonies in the absence of LIF (Figure 7C), and this number was unaltered following deletion of endogenous *Esrrb* alleles. In contrast, *Esrrb* deletion in *Esrrb*^f/fn^+Nanog ESCs produced a decrease in self-renewal efficiency in the presence of LIF (Figure 7C). More impressively, the defining ability of Nanog to promote LIF independence in ESCs was completely demolished by loss of Esrrb (Figure 7C). *Esrrb*^Δ/Δ^+Nanog ESCs primarily formed differentiated colonies in the absence of LIF (Figure 7D). These observations establish that an important component of Nanog function is conferred by its ability to stimulate Esrrb expression in ESCs.

## Discussion

Genome-wide ChIP studies in ESCs have determined that pluripotency factors bind in proximity to one another at target genes (Chen et al., 2008; Kim et al., 2008). This suggests that the expression of these genes is under the combinatorial control of transcription factors of the pluripotency gene regulatory network (GRN) (Ptashne and Gann, 2001). However, it is unclear to what extent changes in the level of a single factor influence the expression of pluripotency GRN targets (Chambers and Tomlinson, 2009). Here we analyzed the effects of altering Nanog levels upon transcription in ESCs. Using the recently developed GeneProf software for integrating published data sets (Halbritter et al., 2012), more than 5,000 genes were confirmed to bind Nanog in at least two independent studies. Surprisingly, however, only 64 genes showed a ≥1.5-fold change in expression 6 hr (p ≤ 0.05) after reinduction of Nanog activity in *Nanog*^−/−^ ESCs. This indicates that the presence of Nanog is not enough for most genes to which Nanog is bound to alter their transcription rates. This may be due to the binding of multiple additional pluripotency transcription factors at these targets, such that loss of Nanog is insufficient to critically diminish the efficiency of combinatorial control of transcription exerted by the remaining factors. Whether combinatorial control may also limit the transcriptional response to changes in the level of other pluripotency transcription factors is a relevant point for the further understanding of the dynamics and robustness of the pluripotency GRN. Modulating the Esrrb level also affects a limited number of genes. Possibly a limited transcriptional response is a common feature of transcription factors that are heterogeneously expressed in ESCs. Previous work (Hall et al., 2009) analyzing the effect of acute Oct4 depletion detected a much greater number of genes showing prompt transcriptional modulation: 2,714 genes showed a ≥1.5-fold change in expression after 5 hr of Oct4 depletion. The radical differences observed between the modulation of Oct4 and Nanog are supported by genetic evidence showing that tight control of Oct4 levels is necessary to maintain the pluripotent state (Niwa et al., 2000), while fluctuations in Nanog confer flexibility to the network (Chambers et al., 2007).

Among the identified Nanog targets, *Esrrb* shows the strongest transcriptional induction. Nanog binds directly at the *Esrrb* locus, recruits RNAPolII to the *Esrrb* promoter, and increases *Esrrb* pre-mRNA levels within 20 min. Esrrb overexpression maintains the ability to form adult chimeras during passage of ESCs at clonal density in the complete absence of gp130 signaling, a function first described for Nanog (Chambers et al., 2003). Another identified Nanog target is Klf4, which, like Klf2 and Tbx3, has also been reported to sustain pluripotency, but without LIF antagonism (Hall et al., 2009; Niwa et al., 2009). Our findings that *Esrrb* and *Klf4* are direct targets of Nanog, coupled with the notion that Esrrb can positively regulate Nanog (van den Berg et al., 2008), identifies Nanog, Esrrb, and Klf4 as acting to stabilize ESC self-renewal through positive feedback (Davidson, 2010; Oliveri et al., 2008).

Given the fact that Esrrb can activate Nanog expression (van den Berg et al., 2008), we excluded the possibility that the effects of Esrrb overexpression were mediated by Nanog by showing that the ability of Esrrb to promote LIF independence is maintained in *Nanog*^−/−^ ESCs. Esrrb shares this ability with Klf2 (Hall et al., 2009). Klf2 overexpression was suggested to allow resistance to differentiation of *Nanog*^+/+^ cells in serum-free medium (Hall et al., 2009). Here we report that Esrrb can also suppress differentiation in serum-free medium; remarkably it can do so in cells lacking Nanog. These results define Esrrb as a potent intrinsic mediator of self-renewal in ESCs, an ability underlined by the capacity of Esrrb to induce LIF independence to a greater extent than Klf4 and with efficiency comparable with that of Nanog. Nonetheless, in the absence of LIF, Esrrb-overexpressing cells formed colonies that had more differentiated margins compared to Nanog-overexpressing colonies. Moreover, doxycycline treatment of animals injected with ESΔN-iNanog, but not ESΔN-iEsrrb, cells produced teratocarcinomas that were almost exclusively composed of EC cells (Table S2). This indicates that Nanog is a stronger suppressor of differentiation than Esrrb, confirming Nanog at the top of the hierarchy of factors able to sustain the undifferentiated state in ESCs.

The reversion of EpiSCs into an ESC-like pluripotent state has been reported by overexpression of several transcription factors including Nanog, Klf4, Klf2, Nr5a, c-Myc, and Stat3 (Guo and Smith, 2010; Guo et al., 2009; Hall et al., 2009; Hanna et al., 2009; Yang et al., 2010). The overexpression of these factors alone is, reportedly, not sufficient to reestablish chimera competency in EpiSCs but must be accompanied by removal of Activin and Fgf (Hall et al., 2009). In addition, of the tested reprogramming factors, Nanog alone is able to revert EpiSCs to chimera competency without the need for additional Gsk3/Erk inhibition (Silva et al., 2009; Theunissen et al., 2011), LIF signaling (Theunissen et al., 2011; Yang et al., 2010), or fibroblast coculture (Hanna et al., 2009). Here we show that Esrrb surpasses Nanog in the efficiency of reprogramming EpiSCs to chimera competent pluripotency. In fact, Esrrb can mediate this effect even in the presence of the complex and supposedly deleterious environment provided by serum and in the absence of LIF. In contrast, Klf4, another Nanog target gene, was unable to revert EpiSCs to ESC pluripotency unless exogenous LIF and inhibitors of Gsk3 and Erk signaling were supplied. These results suggest that Esrrb and Nanog play similar roles during reprogramming. To conclusively consolidate this notion we determined that Esrrb can overcome the strict requirement for Nanog expression during reprogramming (Silva et al., 2009). However, whereas Esrrb induces reprogramming of EpiSCs with greater efficiency than Nanog in wild-type cells, the opposite is true in EpiSCs lacking *Nanog*. The functional overlap between Nanog and Esrrb is not restricted to the conversion between two distinct pluripotent states, since Esrrb can substitute for Nanog during NSC reprogramming by cell fusion. NSCs genetically null for *Nanog* display a lower reprogramming efficiency than wild-type cells in response to Esrrb. Thus, Esrrb and Nanog act cooperatively to induce pluripotency in differentiated cells.

Nanog is required for the formation of the pluripotent epiblast during preimplantation development. A role for Nanog in promoting transition to pluripotency has been also shown in vitro in reprogramming experiments (Silva et al., 2009). The inability of *Nanog*^−/−^ cells to complete transcription-factor-based reprogramming mirrors the phenotype observed in Nanog null embryos, providing a model to study the unique role of Nanog during the acquisition of pluripotency in early development. Here we confirm that Nanog is indeed required for completion of reprogramming but, strikingly, its activity is not unique. Esrrb can also rescue stalled *Nanog*^−/−^ pre-iPSCs. This indicates that future studies should address the possibility that elevated Esrrb expression might also rescue the developmental defects in Nanog null embryos.

These studies demonstrate that Nanog positively regulates *Esrrb* in ESCs. Esrrb is not expressed in EpiSCs (Greber et al., 2010; Han et al., 2010; Osorno and Chambers, 2011; Osorno et al., 2012) and Nanog is expressed at lower levels in EpiSCs compared to ESCs (Han et al., 2010; Osorno and Chambers, 2011). In addition, Esrrb and Nanog show different extinction kinetics during postimplantation development. Esrrb expression is shut off between E5.5 and E6.5, whereas Nanog shows a more gradual downregulation, disappearing at the onset of somitogenesis (Han et al., 2010; Osorno et al., 2012). This may suggest that additional factors are required for Esrrb expression or that the Nanog level required to stimulate Esrrb transcription has a threshold. Future studies should resolve these issues. Moreover, because human ESCs resemble EpiSCs in gene expression (Tesar et al., 2007), it will be of interest to determine the effects of Esrrb expression in human ESCs, particularly as it relates to attempts to establish human ESCs in a “ground state”(Hanna et al., 2010).

Our results reveal a high degree of mutual dependence between Nanog and Esrrb function in ESCs. The ability of Nanog to enhance ESC self-renewal when overexpressed is dependent on Esrrb expression. Conversely, in all our experiments we observed reduced effects of Esrrb overexpression in a *Nanog*^−/−^ background. Nanog and Esrrb proteins interact (Wang et al., 2006) and there is overlap between Esrrb and Nanog targets in ESCs. It will be interesting to see whether some pluripotency GRN targets are sensitive to the combined loss of Nanog and Esrrb.

Finally, our results considerably strengthen the available evidence for the importance of Esrrb in the maintenance of ESC pluripotency. The consequences of Esrrb loss-of-function in ESCs has until now been limited to knockdown experiments (Ivanova et al., 2006). Here we show that Esrrb deletion in ESCs leads to a severely impaired self-renewal ability, reminiscent of the effect of deletion of Nanog (Chambers et al., 2007). Nonetheless, both *Esrrb*^−/−^ and *Nanog*^−/−^ ESCs can be derived. This is in striking contrast to the absolute requirement for Oct4 and Sox2 in pluripotent cells (Avilion et al., 2003; Masui et al., 2007; Nichols et al., 1998; Niwa et al., 2000). Combined with the transcriptional differences in response to Nanog (this study) or Oct4 (Hall et al., 2009) manipulation, this suggests that some pluripotency factors like Oct4 lie at the heart of the housekeeping functions performed by the transcriptional machinery that sustains pluripotency in ESCs, while other factors, such as Nanog, and possibly Esrrb, precisely tune the expression of a limited number of genes that set the conditions for cell fate decisions.

## Experimental Procedures

### ESC Culture

Cells were cultured in GMEMβ-mercaptoethanol/10%FCS/LIF as described (Smith, 1991) or in N2B27 (Ying et al., 2008) supplemented where indicated with PD0325901 (1 μM) and CHIR99021 (3 μM). Colony-forming assays were as described (Chambers et al., 2003).

### Derivation of EpiSCs from ESCs

EpiSCs were derived as described (Guo et al., 2009). EpiSCs were passaged every 5–6 days by incubation with 1× accutase (Sigma, Catalogue no: A 6964) for 5 min, triturated into small clumps of 10–100 cells, neutralized with EpiSC medium, and replated at the appropriate dilution.

### Doxycycline-Inducible Expression

E14Tg2a or TβC44Cre6 cells were transfected with TetO-TdTomato-2a-Hyg^R^-tk and CAG-rtTA-ires-BSD^R^. Clones were screened for high, homogeneous TdTomato expression in doxycycline without continued hygromycin selection and low levels of TdTomato in the absence of doxycycline. An identified cell line was used for FlpE-catalyzed RCME.

### Episomal Reversion of EpiSCs

EpiSCs expressing the large T antigen (E14/T) were transfected with Polyoma *ori*^+^ plasmids using Lipofectamine 2000 (Invitrogen; 11668-019) with 3 μg of pPyCAGgfpIP, pPyCAGDsRedIP, pPyCAGNanogIP, or pPyCAGEsrrbIP. The next day 5 × 10^4^ cells were replated in the presence of puromycin and plates were stained for AP after 7 days. For further analysis, Epi-iPSC colonies were picked and expanded in the absence of puromycin selection.

### Reversion of *Nanog*^−/−^ EpiSCs

5 × 10^4^ EpiΔN-iNanog and EpiΔN-iEsrrb cells were replated in 9 cm dishes in GMEMβ/FCS/LIF +/− doxycycline and plates were stained for AP after 7 days. Epi-iPSC colonies were also picked and expanded in the absence of doxycycline.

### ESC × NSC Fusions

RCNβH(t) NSCs, derived from the RCNβH(t) ESC line, were propagated in NSC medium with FGF/EGF (Conti et al., 2005). 4 × 10^6^ ESCs were fused to 4 × 10^6^ NSCs (Silva et al., 2006), plated in ES medium with appropriate selections (see Supplemental Information), and cultured for 14 or 16 days prior to colony scoring.

## Accession Numbers

The complete analysis pipeline and results for Deep-SAGE and ChIP-Seq data and downstream analysis of microarray data are available in GeneProf under accession number gpXP_000385.

## Supplemental Information

Document S1. Figures S1–S7, Tables S2–S7, and Supplemental Experimental Procedures

Table S1. All Gene Expression Data Relative to Figure 1 and Figure S2
