# Supplementary material for: Esrrb Is a Direct Nanog Target Gene that Can Substitute for Nanog Function in Pluripotent Cells
Source: Cell Stem Cell. 2012 Oct 5;11(4):477–90. doi: 10.1016/j.stem.2012.08.002 (PMC3473361; doi:10.1016/j.stem.2012.08.002)
Supplement: Document S1. Figures S1–S7, Tables S2–S7, and Supplemental Experimental Procedures [file mmc1.pdf]

Cell Stem Cell, volume *11*

Supplemental Information

Esrrb Is a Direct Nanog Target Gene that Can Substitute for Nanog Function in Pluripotent Cells

Nicola Festuccia, Rodrigo Osorno, Florian Halbritter, Violetta Karwacki-Neisius, Pablo Navarro, Douglas Colby, Frederick Wong, Adam Yates, Simon R. Tomlinson, and Ian Chambers

Figures:

Supplementary Figure 1 with legend, **refers to Figure 1**.
Esrrb promptly responds to elevation of Nanog levels in ES cells.

Supplementary Figure 2 with legend, **refers to Figure 1**.
Timecourse microarray analysis of doxycycline induction in ESΔN-iNanog and ESΔN-iEsrrb cells.

Supplementary Figure 3 with legend, **refers to Figure 2**.
Esrrb overexpression confers LIF independent self-renewal at lower doses and independently from Nanog.

Supplementary Figure 4 with legend, **refers to Figure 2**.
Esrrb overexpression confers LIF independent self-renewal in the absence of Nanog.

Supplementary Figure 5 with legend, refers to **Figure 3**.
Stable integration of Esrrb facilitates reversion of EpiSC to Epi-iPS cells.

Supplementary Figure 6 with legend, refers to **Figure 5**.
Esrrb overexpression increases the efficiency of reprogramming by cell fusion and allows generation of stable lines in the absence of Nanog.

Supplementary Figure 7 with legend, refers to **Figure 7**.
Characterisation of Esrrb inducible knockout ES cell lines.

Tables:

Supplementary Table 2 with legend, **refers to Figure 2 and S4**. Esrrb overexpression maintains pluripotency after long-term culture in the absence of LIF.

Supplementary Table 3 with legend, **refers to Figure 3 and 4**. Esrrb induces Epi-iPS cell chimaera forming capacity.

Supplementary Table 4 with legend, **refers to Figure 5 and S6**. In-vitro reprogramming by cell fusion can proceed in the absence of Nanog.

Supplementary Table 5 with legend, **refers to Figure 5**. In-vitro reprogramming by cell fusion can proceed in the absence of Nanog.

Supplementary Table 6 with legend, **refers to Figure 5 and S6**. In-vitro reprogramming by cell fusion can proceed in the absence of Nanog.

Supplementary Table 7 with legend, **refers to Figure 6**. Esrrb induces NS derived iPSΔN-iEssrb chimaera forming capacity.

Supplementary Experimental Procedures

Supplementary References

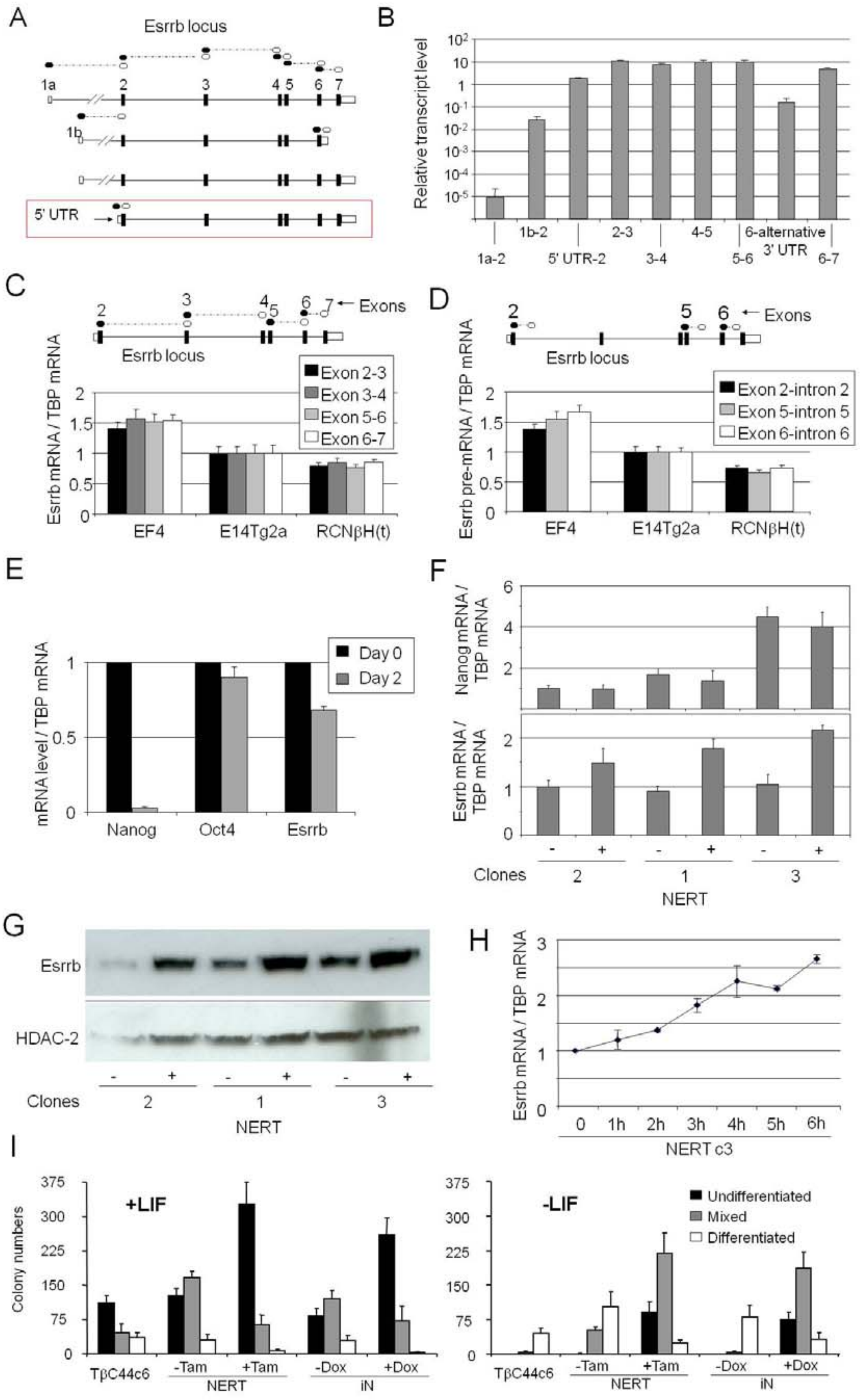
A
Esrrb locus
1a
2
3
4 5
6 7
1b
5' UTR
B
Relative transcript level
1a-2
1b-2
5' UTR-2
2-3
3-4
4-5
5-6
6-alternative 3' UTR
6-7
C
Exons
Esrrb locus
Esrrb mRNA / TBP mRNA
Exon 2-3
Exon 3-4
Exon 5-6
Exon 6-7
EF4
E14Tg2a
RCNβH(t)
D
Exons
Esrrb locus
Esrrb pre-mRNA / TBP mRNA
Exon 2-intron 2
Exon 5-intron 5
Exon 6-intron 6
EF4
E14Tg2a
RCNβH(t)
E
mRNA level / TBP mRNA
Day 0
Day 2
Nanog
Oct4
Esrrb
F
Nanog mRNA / TBP mRNA
Esrrb mRNA / TBP mRNA
Clones
NERT
G
Esrrb
HDAC-2
Clones
NERT
H
Esrrb mRNA / TBP mRNA
0
1h
2h
3h
4h
5h
6h
NERT c3
I
Colony numbers
+LIF
-LIF
Undifferentiated
Mixed
Differentiated
TβC44c6
-Tam
+Tam
NERT
-Dox
+Dox
iN

Supplementary Figure 1 (Relates to Figure 1): Esrrb promptly responds to elevation of Nanog levels in ES cells.

A: Schematic representation of the structure of four mouse Esrrb transcript variants identified in EST databases; black boxes: exons; white boxes: 5' and 3' untranslated regions (UTRs); solid lines: introns; black and white circles connected by dashed lines: forward and reverse primers used in panel B. **B**: Quantitative assessment of relative transcript levels using primers to amplify the indicated exon-exon junctions. Error bars: standard deviation (n=3). **C, D**: Esrrb mRNA (C) and pre-mRNA (D) levels in EF4, E14Tg2a and RCNβH(t) ES cells. Relative expression levels measured using the indicated primer pairs. Error bars: standard deviation of the mean (n=5). **E**: Esrrb, Nanog and Oct4 mRNA levels in RCNβH cells in which loss of Nanog expression is induced by tamoxifen treatment. Error bars: standard deviation (n=2 day0; n=6 day2). **F**: Nanog and Esrrb expression in 3 independent ESΔN-NERT (NERT) lines cultured in the presence or absence of tamoxifen for 24 hours. Error bars are the standard deviation of the 3 technical replicates of one experiment. **G**: Immunoblot analysis of Esrrb and HDAC-2 protein expression in 3 ESΔN-NERT clones (NERT) cultured in the presence or absence of tamoxifen for 24 hours. **H**: Kinetics of Esrrb mRNA upregulation in ESΔN-NERTc3 cells stimulated with tamoxifen for the indicated number of hours. Error bars: standard deviations of 3 independent experiments. **I**: Number and type of colonies formed 7 days after clonal density plating of TβC44Cre6, ESΔN-NERT or ESΔN-iNanog cells in the presence or absence of LIF, with or without doxycycline or tamoxifen. Error bars: standard deviation (n=3).

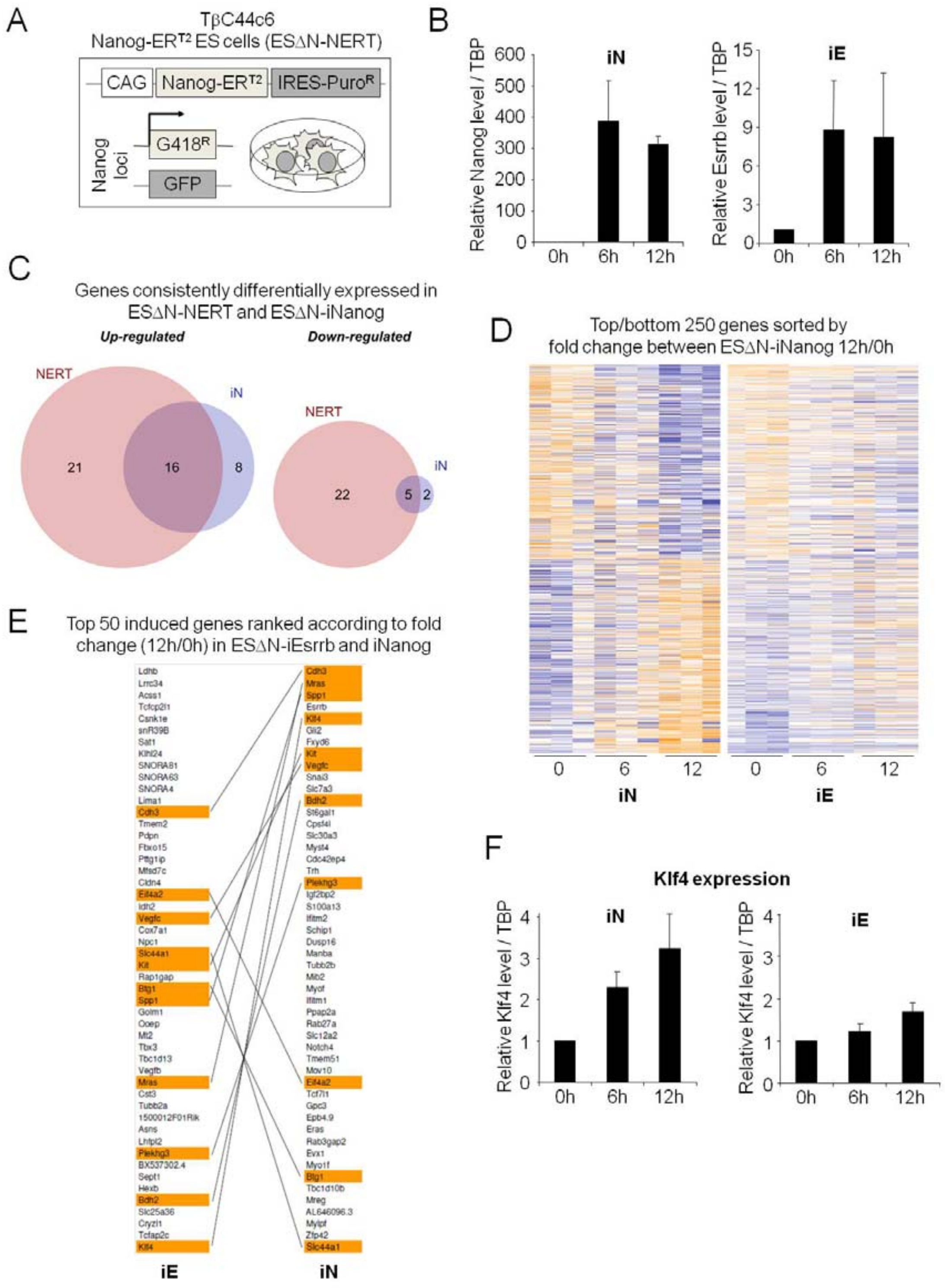
A
TβC44c6
Nanog-ER^{T2} ES cells (ESΔN-NERT)
CAG
Nanog-ER^{T2}
IRES-Puro^R
Nanog loci
G418^R
GFP
B
iN
Relative Nanog level / TBP
0h 6h 12h
iE
Relative Esrrb level / TBP
0h 6h 12h
C
Genes consistently differentially expressed in ESΔN-NERT and ESΔN-iNanog
Up-regulated
Down-regulated
NERT
iN
21
16
8
22
5
2
D
Top/bottom 250 genes sorted by fold change between ESΔN-iNanog 12h/0h
0 6 12 0 6 12
iN
iE
E
Top 50 induced genes ranked according to fold change (12h/0h) in ESΔN-iEsrrb and iNanog
iE
iN
F
Klf4 expression
Relative Klf4 level / TBP
0h 6h 12h

Supplementary Figure 2 (Relates to Figure 1): Timecourse microarray analysis of doxycycline induction in ESΔN-iNanog and ESΔN-iEsrrb cells.

A: Schematic representation of the genetic manipulations used to make ESΔN-NERT cells. **B**: Nanog and Esrrb mRNA expression in ESΔN-iNanog (iN) and ESΔN-iEsrrb (iE) lines cultured in the presence of doxycycline for the indicated times. Error bars: standard deviations of 3 independent experiments. **C**: Intersection of significantly (fold change ≥ 1.5; p≤0.05) up- or downregulated genes identified by doxycycline stimulation of ESΔN-iNanog (iN) for 12 h and tamoxifen stimulation of ESΔN-NERT (NERT) for 6h. **D**: Expression levels of the top 250 and bottom 250 genes that were differentially expressed in ESΔN-iNanog cells after 12h of doxycycline treatment, compared at the indicated times (hours) in ESΔN-iNanog (iN) or ESΔN-iEsrrb (iE) cells. Colours; expression above average (yellow) and below average (blue). **E**: Comparison of the 50 Nanog and Esrrb bound genes most upregulated in ESΔN-iNanog (iN) and ESΔN-iEsrrb (iE) after a 12h doxycycline treatment. Genes were ranked according to fold induction in ESΔN-iNanog or ESΔN-iEsrrb respectively. Shared targets are highlighted (orange) and connected by lines. **F**: Klf4 expression in ESΔN-iNanog (iN) and ESΔN-iEsrrb (iE) cells treated with doxycycline as indicated. Error bars: standard deviations of 3 independent experiments.

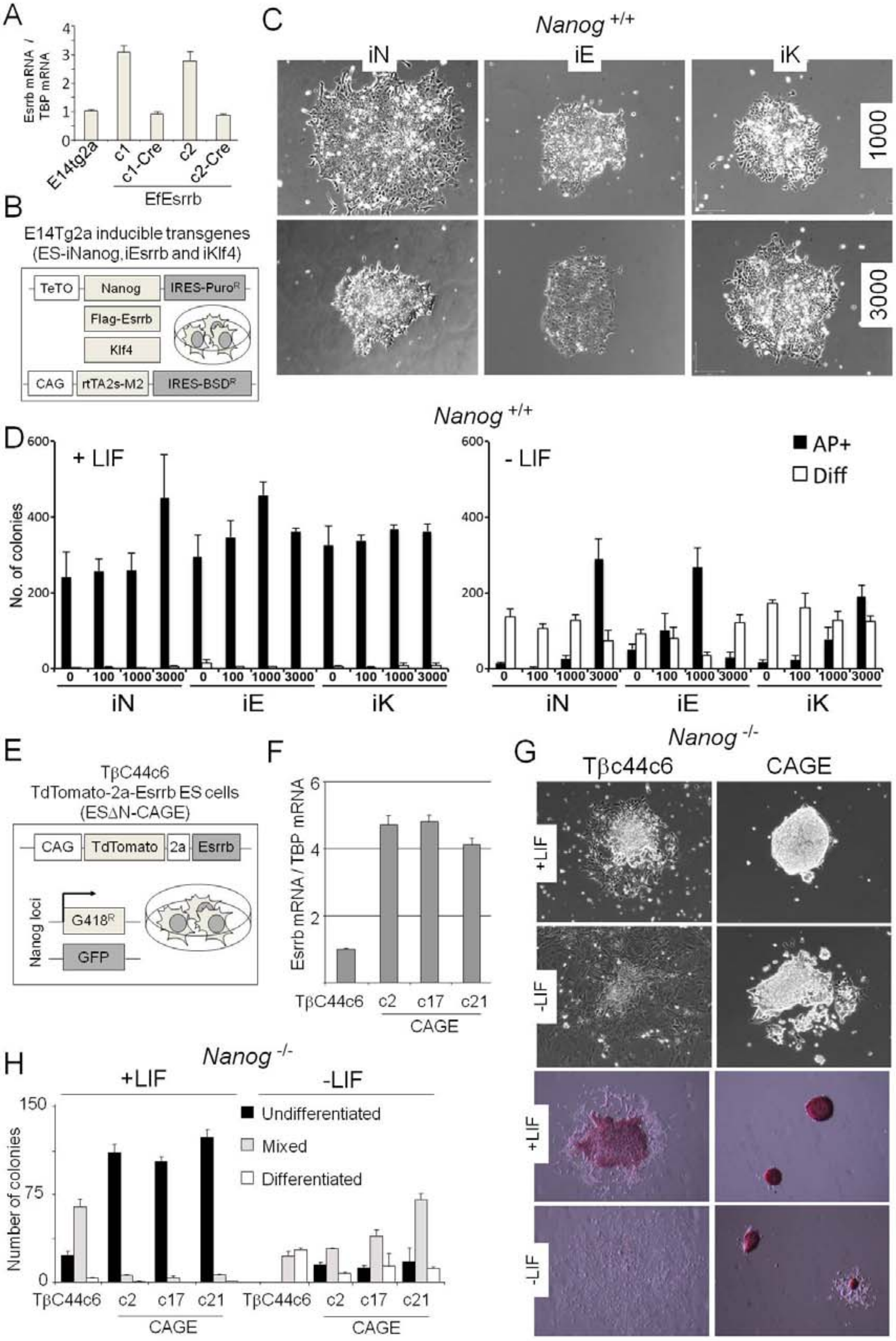
A
Esrrb mRNA / TBP mRNA
E14tg2a
c1
c1-Cre
c2
c2-Cre
EfEsrrb
B
E14Tg2a inducible transgenes
(ES-iNanog,iEsrrb and iKlf4)
TeTO
Nanog
IRES-Puro^R
Flag-Esrrb
Klf4
CAG
rtTA2s-M2
IRES-BSD^R
C
Nanog +/+
iN
iE
iK
1000
3000
D
+ LIF
- LIF
AP+
Diff
No. of colonies
iN
iE
iK
E
TβC44c6
TdTomato-2a-Esrrb ES cells
(ESΔN-CAGE)
CAG
TdTomato
2a
Esrrb
Nanog loci
G418^R
GFP
F
Esrrb mRNA / TBP mRNA
TβC44c6
c2
c17
c21
CAGE
G
Nanog -/-
Tβc44c6
CAGE
+LIF
-LIF
H
Nanog -/-
+LIF
-LIF
Undifferentiated
Mixed
Differentiated
Number of colonies
TβC44c6
c2
c17
c21
CAGE

Supplementary Figure 3 (Relates to Figure 2). Esrrb overexpression confers LIF independent self-renewal at lower doses and independently from Nanog.

A: Esrrb transcript levels in EfEsrrb ES cells before and after treatment with Cre. Error bars are the standard deviation of the 3 technical replicates of one experiment. **B**: Schematic representation of the genetic manipulations used to make ES-iNanog, ES-iEsrrb or ES-iKlf4 cells. **C**: Colony morphology of ES-iNanog (iN), ES-iEsrrb (iE) or ES-iKlf4 (iK) cells plated at clonal density and cultured in 1000 or 3000 ng/ml doxycycline and in the absence of LIF for 8 days. **D:** Numbers of alkaline phosphatase colonies scored after plating ES-iNanog (iN), ES-iEsrrb (iE) or ES-iKlf4 (iK) cells at clonal density in the presence or absence of LIF and with the indicated doxycycline concentration for 7 days. Error bars: standard deviation (n=3). **E:** Schematic representation of the genetic manipulations performed on TβC44Cre6 TdTomato-2a-Esrrb ES cells (ESΔN-CAGE). **F**: Transcript levels of Esrrb in TβC44Cre6 and ESΔN-CAGE (CAGE) cells. Error bars are the standard deviation of the 3 technical replicates of one experiment. **G**: Top panels, colony morphology of TβC44Cre6 and ESΔN-CAGE (CAGE) cells plated at clonal density and cultured in the presence or in the absence of LIF for 8 days. Bottom panels, alkaline phosphatase staining of colonies. **H**: Numbers of alkaline phosphatase colonies scored in 3 independent ESΔN-CAGE (CAGE) clones and parental TβC44Cre6 ES cells after plating at clonal density and culture in the presence or absence of LIF for 8 days. Error bars: standard deviation (n=3).

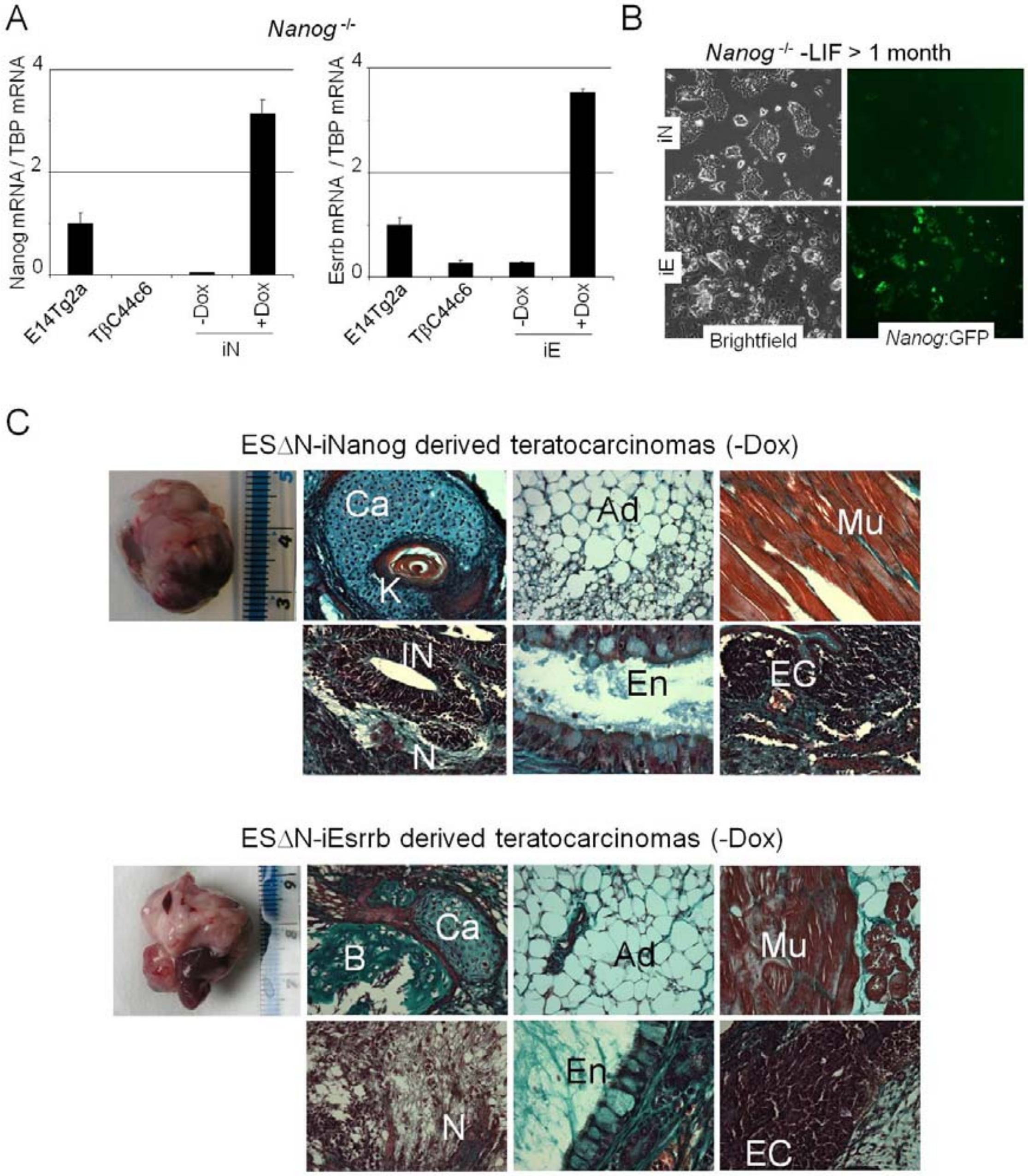
A
Nanog -/-
Nanog mRNA / TBP mRNA
Esrrb mRNA / TBP mRNA
4
2
0
E14Tg2a
TβC44c6
-Dox
+Dox
iN
iE
B
Nanog -/- -LIF > 1 month
iN
iE
Brightfield
Nanog:GFP
C
ESΔN-iNanog derived teratocarcinomas (-Dox)
Ca
K
Ad
Mu
IN
N
En
EC
ESΔN-iEsrrb derived teratocarcinomas (-Dox)
B
Ca
Ad
Mu
N
En
EC

Supplementary Figure 4 (Relates to Figure 2). Esrrb overexpression confers LIF independent self-renewal in the absence of Nanog.

A: Transcript levels of Nanog in ESΔN-iNanog (iN) cells and Esrrb in ESΔN-iEsrrb (iE) cells cultured in the presence or in the absence of doxycycline. Error bars: standard deviation of gene expression measured in two independent clones (ESΔN-iNanog or ESΔN-iEsrrb) or three independent experiments (E14Tg2a, TβC44Cre6). **B**: Morphology and *Nanog*:GFP expression of ESΔN-iNanog (iN) and ESΔN-iEsrrb (iE) cells cultured in the presence of doxycycline and LIF antagonist (hLIF-05) for more than one month. **C**: Representative teratocarcinomas recovered after injection under the kidney capsule of ESΔN-iNanog or ESΔN-iEsrrb ES cells that had been cultured in the presence of LIF antagonist for more than one month, along with examples of tissue types used for scoring germ layer contribution. Ca: cartilage; K: keratinised epithelium; En: Endoderm; N: mature neural tissue; IN: Immature neural tissue; B: Bone; Mu: Skeletal muscle; Ad: Adipose tissue; EC, embryonal carcinoma.

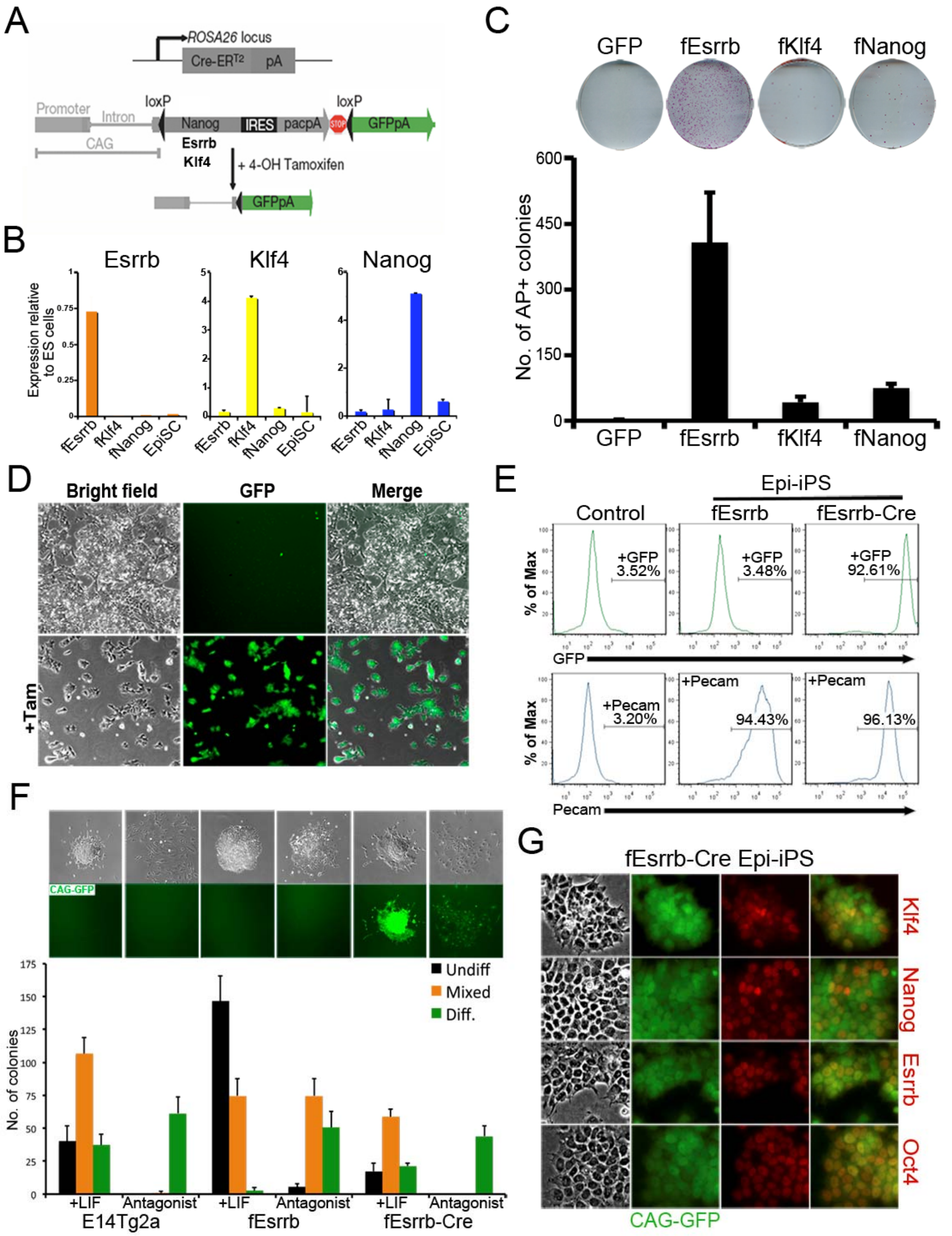
A
ROSA26 locus
Cre-ERT2
pA
Promoter
Intron
loxP
Nanog
IRES
pacpA
STOP
loxP
GFPpA
CAG
Esrrb
Klf4
+ 4-OH Tamoxifen
GFPpA
B
Esrrb
Klf4
Nanog
Expression relative to ES cells
fEsrrb
fKlf4
fNanog
EpiSC
C
GFP
fEsrrb
fKlf4
fNanog
No. of AP+ colonies
D
Bright field
GFP
Merge
+Tam
E
Epi-iPS
Control
fEsrrb
fEsrrb-Cre
% of Max
+GFP 3.52%
+GFP 3.48%
+GFP 92.61%
GFP
+Pecam 3.20%
+Pecam 94.43%
+Pecam 96.13%
Pecam
F
CAG-GFP
Undiff
Mixed
Diff.
No. of colonies
+LIF
Antagonist
E14Tg2a
fEsrrb
fEsrrb-Cre
G
fEsrrb-Cre Epi-iPS
Klf4
Nanog
Esrrb
Oct4
CAG-GFP

Supplementary Figure 5 (Relates to Figure 3). Stable integration of Esrrb facilitates reversion of EpiSC to Epi-iPS cells.

A: Schematic diagram of the RC EpiSC line and the Cre revertible vectors used for stable integration of Nanog, Klf4 or Esrrb. **B**: mRNA expression of Nanog, Klf4 and Esrrb in RC EpiSC, fEsrrb (RC + floxed-Esrrb), fKlf4 (RC + floxed-Klf4) and fNanog (RC + floxed-Nanog) cultured in EpiSC media. Error: Standard deviation of the mean in at least 2 independent experiments. **C:** Number of alkaline phosphatase positive colonies observed 7 days after replating 2×10^5 EpiSC stably transfected with Cre revertible GFP, Nanog, Klf4 and Esrrb vectors in 2i/LIF/N2B27. Error: Standard deviation of the mean in 3 independent experiments. Representative AP stained plates are shown on top of the graph. **D**: Esrrb induced Epi-iPS cells were treated with 1μM of Tamoxifen (Sigma) for 24h to excise the floxed Esrrb vector. Successful Cre-mediated excision is reported by ubiquitous eGFP expression. **E**: Flow cytometry analysis of GFP (top panels) and Pecam (bottom panels) expression on Epi-iPS before and after treatment with Tamoxifen for 24h. **F**: Top panel: morphology in E14Tg2a, fEsrrb and f-Esrrb-Cre reverted ES cells plated at clonal density in complete ES medium (FCS) in the presence of LIF or LIF antagonist. Bottom panel: Quantification of the clonal assay; error bars are the standard deviation of the mean in 3 independent experiments. **G**: Immunohistochemistry for Klf4, Esrrb, Nanog and Oct4 proteins in fEsrrb-Cre reverted ES cells cultured in FCS/LIF/GMEMβ.

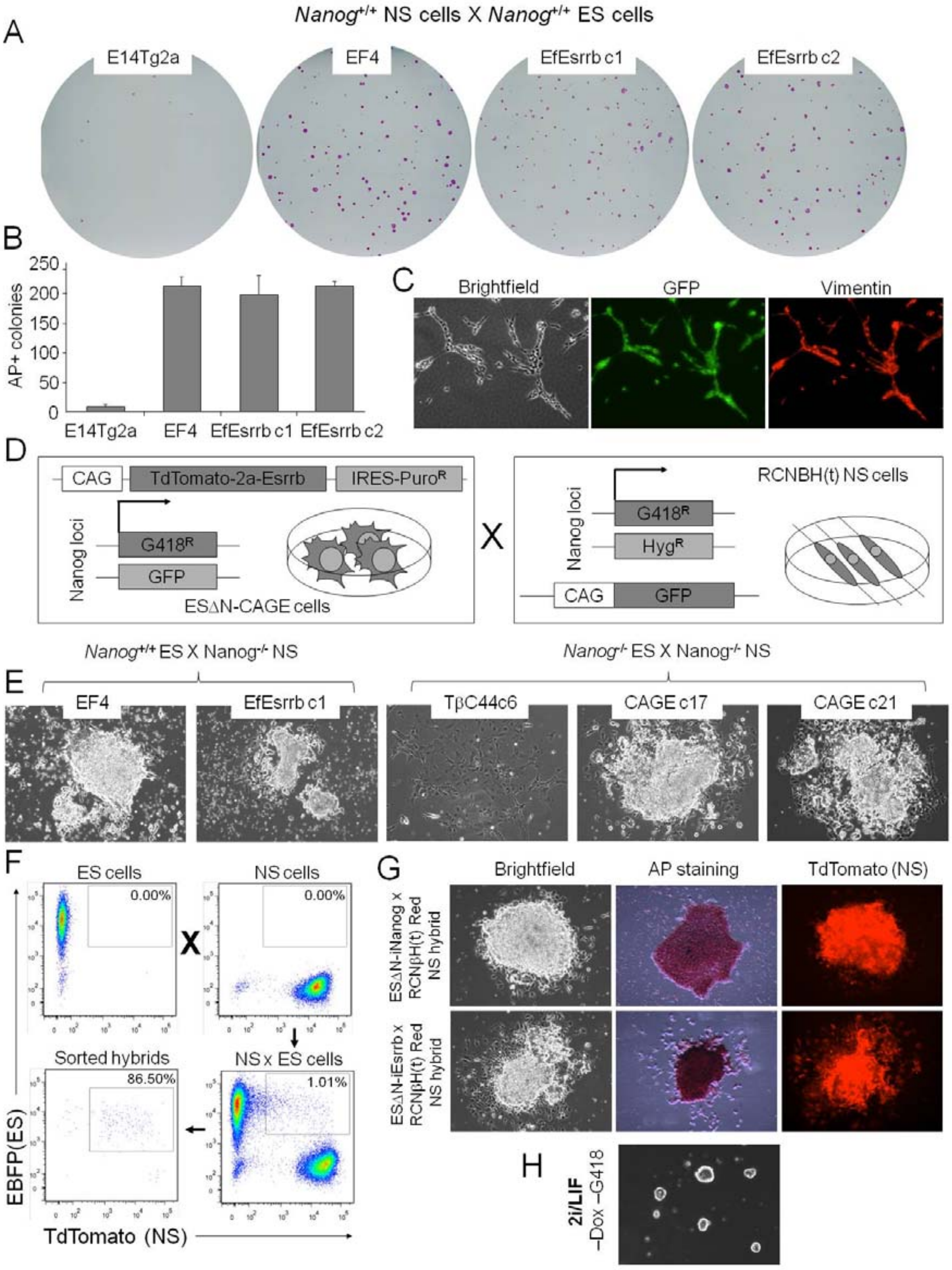
Nanog+/+ NS cells X Nanog+/+ ES cells
A
E14Tg2a
EF4
EfEsrrb c1
EfEsrrb c2
B
AP+ colonies
250
200
150
100
50
0
E14Tg2a
EF4
EfEsrrb c1
EfEsrrb c2
C
Brightfield
GFP
Vimentin
D
CAG
TdTomato-2a-Esrrb
IRES-PuroR
Nanog loci
G418R
GFP
ESΔN-CAGE cells
X
RCNBH(t) NS cells
Nanog loci
G418R
HygR
CAG
GFP
Nanog+/+ ES X Nanog-/- NS
Nanog-/- ES X Nanog-/- NS
E
EF4
EfEsrrb c1
TβC44c6
CAGE c17
CAGE c21
F
ES cells
0.00%
X
NS cells
0.00%
Sorted hybrids
86.50%
NS x ES cells
1.01%
EBFP(ES)
TdTomato (NS)
G
Brightfield
AP staining
TdTomato (NS)
ESΔN-iNanog x RCNβH(t) Red NS hybrid
ESΔN-iEsrrb x RCNβH(t) Red NS hybrid
H
2i/LIF
–Dox –G418

Supplementary Figure 6 (relates to Figure 5). Esrrb overexpression increases the efficiency of reprogramming by cell fusion and allows generation of stable lines in the absence of Nanog.

A: Representative plates from ES x NS fusion experiments performed using the indicated lines (all cells are *Nanog*$^{+/+}$, EF4 ES cells overexpress Nanog; NS cells are derived from E14/T cells). ES x NS hybrids were cultured for 12 days in puromycin and neomycin selection prior to staining for alkaline phosphatase. **B**: AP positive colony numbers/million NS fused observed in the above experiment; error bars are the standard deviation (n=4). **C**: Immunofluorescence analysis for vimentin and GFP in RCNβH(t) NS cells. **D**: Schematic representation of the two lines used in the fusion experiments: TβC44Cre6 derived ESΔN-CAGE cells and RCNβH(t) NS cells. **E**: Morphology of hybrid colonies formed by fusion of RCNβH(t) NS cells with the indicated ES cell lines (EF4 cells overexpress Nanog). Hybrids were cultured for 15 days; puromycin was applied after 1 day; hygromycin application was delayed until day 4 to allow reprogramming of the NS genome. **F**: ESΔN-iNanog or ESΔN-iEsrrb cells were transfected with a CAG-EBFP expression vector and clones showing high EBFP expression were selected. ESΔN-iNanog Blue or ESΔN-iEsrrb Blue x RCNβH(t) Red NS primary hybrids were purified by FACS sorting 24h after fusion on the basis of EBFP and TdTomato expression. FACS plots showing TdTomato expression in NS cells, EBFP in ES cells before fusion, and coincident TdTomato and EBFP expression in hybrids one day after fusion and in the sorted population. **G**: TdTomato expression (marking the NS cell genome) in ESΔN-iNanog or ESΔN-iEsrrb cells x RCNβH(t) Red NS hybrid lines cultured in ES cell medium confirms productive fusion. Central panels show alkaline phosphatase staining of colonies. **H**: ESΔN-iEsrrb x RCNβH(t) NS hybrid after three passages in N2B27/2i/LIF.

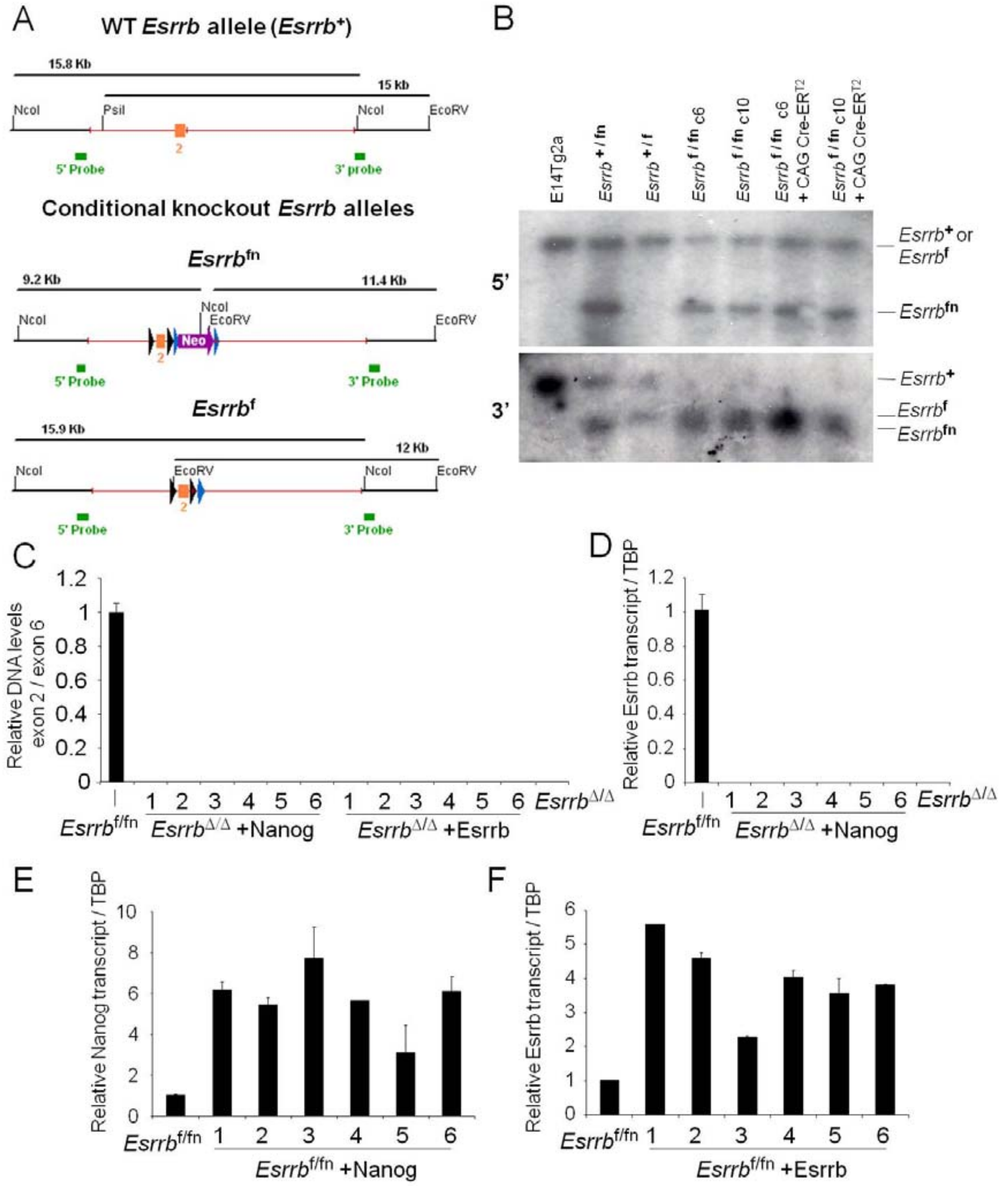
A
WT Esrrb allele (Esrrb+)
15.8 Kb
15 kb
NcoI
PsiI
NcoI
EcoRV
2
5' Probe
3' probe
Conditional knockout Esrrb alleles
Esrrbfn
9.2 Kb
11.4 Kb
NcoI
NcoI
EcoRV
EcoRV
Neo
5' Probe
3' Probe
Esrrbf
15.9 Kb
12 Kb
NcoI
EcoRV
NcoI
EcoRV
5' Probe
3' Probe
B
E14Tg2a
Esrrb +/fn
Esrrb +/f
Esrrb f/fn c6
Esrrb f/fn c10
Esrrb f/fn c6 + CAG Cre-ERT2
Esrrb f/fn c10 + CAG Cre-ERT2
5'
3'
Esrrb+ or Esrrbf
Esrrbfn
Esrrb+
Esrrbf
Esrrbfn
C
Relative DNA levels exon 2 / exon 6
1.2
1
0.8
0.6
0.4
0.2
0
Esrrbf/fn
1 2 3 4 5 6
EsrrbΔ/Δ +Nanog
1 2 3 4 5 6
EsrrbΔ/Δ +Esrrb
EsrrbΔ/Δ
D
Relative Esrrb transcript / TBP
1.2
1
0.8
0.6
0.4
0.2
0
Esrrbf/fn
1 2 3 4 5 6
EsrrbΔ/Δ +Nanog
EsrrbΔ/Δ
E
Relative Nanog transcript / TBP
10
8
6
4
2
0
Esrrbf/fn
1 2 3 4 5 6
Esrrbf/fn +Nanog
F
Relative Esrrb transcript / TBP
6
5
4
3
2
1
0
Esrrbf/fn
1 2 3 4 5 6
Esrrbf/fn +Esrrb

Supplementary Figure 7 (relates to Figure 7). Characterisation of Esrrb inducible knockout ES cell lines.

A: Schematic representation of the wild type and conditional knockout *Esrrb* alleles. Homology arms are shown in red. The restriction enzyme sites used for Southern blot analysis are shown, along with the expected size of the genomic DNA fragment generated by digestion. loxP sites are shown as black triangles. FRT sites are blue triangles. **B**: Southern blot analysis of $Esrrb^{+/fn}$, $Esrrb^{+/f}$ and $Esrrb^{f/fn}$ lines generated after the first and second targeting events from E14Tg2a ES cells. Two separate double targeted $Esrrb^{f/fn}$ lines are shown before and after stable integration of a CRE-ER^{T2} ubiquitously expressed transgene, demonstrating that excision does not occur in the absence of tamoxifen treatment. **C**: Quantitative PCR analysis of the relative levels of *Esrrb* exon 2 to *Esrrb* exon 6 in $Esrrb^{f/fn}$, $Esrrb^{\Delta/\Delta}$, $Esrrb^{\Delta/\Delta}$ +Nanog and $Esrrb^{\Delta/\Delta}$ +Esrrb ES cells. Error bars are the standard deviation of the 3 technical replicates of one experiment. **D**: Esrrb transcript levels in $Esrrb^{f/fn}$, $Esrrb^{\Delta/\Delta}$, and $Esrrb^{\Delta/\Delta}$ +Nanog cells. Error bars are the standard deviation of the 3 technical replicates of one experiment. **E**: Nanog transcript levels in $Esrrb^{f/fn}$ and $Esrrb^{f/fn}$ + Nanog ES cells. Error bars are the standard deviation of the 3 technical replicates of one experiment. **F**: Esrrb transcript levels in $Esrrb^{f/fn}$ and $Esrrb^{f/fn}$ +Esrrb ES cells. Error bars are the standard deviation of the 3 technical replicates of one experiment.

	Animal number	Doxycycline	Tumour size (mm x mm)	Ectoderm	Mesoderm	Endoderm	EC
ESΔN-iNanog	2	+	11 x 13				√
	3	+	18 x 24	√			√
	4	+	7 x 5				√
	5	+	10 x 15	√	√		√
	2	-	9 x 6	√	√	√	
	3	-	17 x 17	√	√		√
	4	-	18 x 20	√	√	√	√
	5	-	1 x 1	√	√		

	Animal number	Doxycycline	Tumour size (mm xmm)	Ectoderm	Mesoderm	Endoderm	EC
ESΔN-iEsrrb	1	+	20 x 22	√	√		√
	2	+	15 x 16	√	√	√	√
	3	+	11 x 16	√	√	√	√
	4	+	3 x 3	√	√	√	
	1	-	16 x 17	√	√	√	√
	2	-	10 x 12	√	√	√	
	3	-	19 x 22	√	√	√	
	4	-	13 x 15	√	√	√	
	5	-	No tumour				

Supplementary Table 2 (Refers to Figure 2 and S4). Esrrb overexpression maintains pluripotency after long-term culture in the absence of LIF.

ESΔN-iNanog or ESΔN-iEsrrb ES cells were cultured for more than one month in the presence of LIF antagonist and doxycycline. The table shows tumour size and tissue contribution observed after injection of ESΔN-iNanog or ESΔN-iEsrrb cells in the kidney capsule of congenic animals fed or not fed doxycycline. Animals were sacrificed and tumours dissected exactly one month after injection.

Cell line	No. of born mice	Chimaeras	% of chimaeras
E14/T (Esrrb C3) *Nanog*$^{+/+}$	15	5	33%
TNG/T (Esrrb C8) *Nanog*$^{+/GFP}$	7	2	28.6%
Epi-iPSΔN-iEsrrb C1 Nanog $^{-/GFP}$	24	3	12.5%
Epi-iPSΔN-iEsrrb C1 Nanog $^{-/GFP}$	18	2	11.1%

Supplementary Table 3 (relates to Figure 3 and 4). Esrrb induced Epi-iPS cell chimaera forming capacity. The number of adult chimaeras obtained from four independent blastocyst injections.

RCNβH(t) NS X	Hybrids/10^6 cells fused	RCNβH(t) NS X	Hybrids/10^6 cells fused
Nanog (EF4)	453	NS RCNβH(t)	0
EfEsrrb c1	133	TβC44c6	0
TβC44c6	0	TβC44	1
ESΔN-CAGE c17	1.6	ESΔN-CAGE c17	4.25
ESΔN-CAGE c21	2.5	ESΔN-CAGE c21	32.5

Supplementary Table 4 (relates to Figure 5 and S6). In-vitro reprogramming by cell fusion can proceed in the absence of Nanog. The number of AP positive colonies observed after fusion of RCNβH(t) NS cells with the indicated ES cell lines in two independent experiments (left panel - right panel). Hybrids were cultured for 14 days; puromycin and hygromycin selections were applied from day 1 and day 4 respectively.

ESΔN-iNanog or ESΔN-iEsrrb
x
RCNβH(t) Red NS plating

Fusion	Number of NS cells fused	AP^+ Colonies (± st dev.)	AP^+ colonies/10^6 cells fused (± st dev.)
ESΔN-iNanog +Dox	4,000,000	**1215** (± 588)	**303.7** (± 147.1)
ESΔN-iNanog -Dox	4,000,000	6 (± 8)	1.6 (± 2.1)
ESΔN-iEsrrb +Dox	4,000,000	**99** (± 76)	**24.8** (± 19.1)
ESΔN-iEsrrb -Dox	4,000,000	17 (± 27)	4.2 (± 6.7)

Supplementary Table 5 (relates to Figure 5). In-vitro reprogramming by cell fusion can proceed in the absence of Nanog. Reprogramming efficiency observed after fusing ESΔN-iNanog or ESΔN-iEsrrb cells to RCNβH(t) Red NS cells. The number of alkaline phosphatase positive colonies was scored after culturing hybrids for 16 days in blasticidin/hygromycin selection and in the presence or absence of doxycycline. Error: standard deviations of three independent experiments.

ES∆N-iNanog or ES∆N-iEsrrb
x
RCNβH(t) Red NS

Sorted hybrids plating

Fusion	Number of primary Hybrids	AP+ colonies	Purity	Reprogramming frequency
ES∆N-iNanog replica 1	24,000	360	85%	1.76%
ES∆N-iNanog replica 2	14,000	222	82%	1.93%
Total				**1.82%**
Fusion	Number of primary Hybrids	AP+ colonies	Purity	Reprogramming frequency
ES∆N-iEsrrb replica 1	7659	6	62%	0.126%
ES∆N-iEsrrb replica 2	8763	3	45%	0.076%
ES∆N-iEsrrb replica 3	4872	2	62%	0.066%
ES∆N-iEsrrb replica 4	8042	1	66%	0.019%
ES∆N-iEsrrb replica 5	7359	4	51%	0.107%
ES∆N-iEsrrb replica 6	9946	3	50%	0.06%
Total				**0.074%**

Supplementary Table 6 (relates to Figure 5 and S6). In-vitro reprogramming by cell fusion can proceed in the absence of Nanog. The number of alkaline phosphatase positive colonies observed after culturing for 16 days in blasticidin, hygromycin and doxycycline sorted primary hybrids from cell fusions between ESΔN-iNanog Blue or ESΔN-iEsrrb Blue cells and RCNβH(t) Red NS cells. The table shows the sorting purity, the numbers of primary hybrids plated and AP positive colonies counted in each independent experiment, along with the efficiency of NS cells reprogramming.

Cell line	No. of embryos	Midgestation Chimaeras (E11.5)	% of chimaeras	Somatic chimaerism (E11.5)
iPSΔN-iEsrrb C1	18	11	55.6%	3 high; 2 medium; 3 low (3 high contribution abnormal chimaeras)
iPSΔN-iEsrrb C1	19	8	45.5%	2 high; 3 medium; 2 low (1 high contribution abnormal chimaera)

Supplementary Table 7 (relates to Figure 6). Esrrb induces NS derived iPSΔN-iEssrb chimaera forming capacity. Quantification of midgestation chimaeras (E11.5) obtained from two blastocyst injections done with iPSΔN-iEsrrb C1 cells. Contribution was judged by the proportion of TdTomato positive cells present in the chimaeras (High>66.6%; 66.6%<Medium>33.3%; Low<33.3%).

Supplementary Experimental Procedures

Kidney capsule grafts, recovery and processing

Engraftments were performed as described elsewhere (Tam, 1990). 4 weeks after the graft was performed, the mice were sacrificed and the kidneys removed in PBS. After imaging, the tumour and kidney were fixed in 4% paraformaldehyde (PFA) between 1-7 days depending on tumour size. After fixation, the kidneys were dehydrated through ethanol series, cleared in xylene and embedded in paraffin wax before being sectioned in a microtome.

Derivation of EpiSC from ES cells

EpiSC were derived in vitro from ES cells as described (Guo et al., 2009) and passaged by incubation with 1x accutase (Sigma, Catalogue no: A 6964) for 5min and then triturated into small clumps of 10–100 cells, neutralised with EpiSC medium and replated at the appropriate dilution. Subsequent passages were performed every 5-6 days.

Derivation of *Esrrb*$^{\Delta/\Delta}$ ES cells.

Esrrb$^{f/fn}$ ES cells, continuously kept in GMEMβ/FCS/LIF and zeocin selection to ensure Cre-ER^{T2} expression, were passaged once in the presence of 1 μM PD 0325901(Axon Medchem). Cells were replated at clonal density in 10 cm dishes and after 16 hours 1 μM tamoxifen (Sigma) was added to the culture medium. After 1 hour of tamoxifen treatment, plates were washed with PBS and cells cultured for additional 2 weeks in FCS/LIF/GMEMβ supplemented with 1μM PD 0325901. Morphologically distinguishable *Esrrb*$^{\Delta/\Delta}$ colonies

were picked and expanded in the presence of PD 0325901. After expansion, *Esrrb*$^{\Delta/\Delta}$ cells were further cultured in FCS/LIF/GMEMβ.

Detection of *Esrrb* exon 2 excision by quantitative PCR on genomic DNA.

Genomic DNA from *Esrrb*$^{\Delta/\Delta}$ or *Esrrb*$^{f/fn}$ cells was isolated using DNeasy minikits (Qiagen). Real-time RT–PCR reactions were performed in triplicate in 384-wells plates with a 480 LightCycler (Roche) using the LightCycler 480 Probes Master mix (Roche). 100 ng of genomic DNA diluted in 5 microliters of water were used per reaction. For each sample two sets of reactions using primers and Universal Probe Library (UPL)(Roche) probes binding to *Esrrb* exon2 or exon 6 were performed. For each sample, *Esrrb* exon 2 copy numbers were normalised against exon 6 copy numbers, to correct for errors in the quantity of genomic DNA used per reaction. Exon 2/exon 6 ratios are shown relative to untreated *Esrrb*$^{f/fn}$ cells. Standard curves of all primers were performed to check for efficient amplification (above 85%). PCR primer sequences and UPL numbers are available on request.

RNA isolation and quantitative real-time RT-PCR

Total RNA from cultured cells was isolated using the RNeasy microkit or minikit (Qiagen), and performing on-column digestion with DNase I (Qiagen). Reverse transcription reactions were performed on 30 ng-2ug of total RNA in a final volume of 20µL with 100U of SuperScriptIII (Invitrogen) and 200ng random hexamers (Invitrogen) or 250ng of oligodT$^{12-18}$ (Invitrogen) primers at 42°C for 60 minutes. Real-time RT–PCR reactions were performed in triplicate in 384-wells plates with a 480 LightCycler (Roche) using LightCycler 480 SYBR Green I Master (Roche). Five microliters of cDNA or immunoprecipitated chromatin were

used per reaction. Standard curves of all primers were performed to check for efficient amplification (above 85%), and all melting curves were generated to verify production of single DNA species with each primer pair. PCR primer sequences are available on request. Values for each gene were normalised to expression of TATAbox Binding Protein (TBP) or 28S rRNA (For flavopiridol treatment experiments).

Immunohistochemistry

Cultured cells were fixed in 4% PFA for 10 min at RT. Permeabilisation was done with PBS/0.1%v/v TritonX100 for 15 min at RT. Blocking was performed as above, but for 30 min at room temperature. Primary antibodies were diluted in blocking buffer to the working concentrations indicated below and applied for 1-2 h at RT or overnight at 4 °C. After three washes in PBS/0.1%v/v TritonX100, secondary antibodies were diluted to 2μg/ml in blocking buffer and applied for 1 h at RT. The cells were washed at least three times in PBS/0.1%v/v TritonX100 and in selected cases nuclei were stained with 4',6-diamidino-2-phenylindole (DAPI). Primary antibodies were used at the following concentrations: Nanog (ab14959, Abcam), 2.5ug/ml; in-house made anti-Nanog rabbit polyclonal, 1.2-0.25 μg/ml (Chambers et al., 2007); Oct4 (sc-5279, Santa Cruz Biotechnology), 1 μg/ml; Sox2 (sc 17320, Santa Cruz Biotechnology), 1μg/ml; Esrrb (PP-H6705-00, Persaeus Proteomics), 1 μg/ml; TuJ (MMS-435P,Covant), 1 μg/ml.

Imaging

Images were captured using Volocity (Improvision) software in an Olympus IX51 (for cultured cells), or an Olympus BX61 (for teratocarcinoma sections). Image processing was

performed using Adobe Photoshop software. ESΔN-NERT cells stained for Nanog were imaged using a Leica DM IRE2 inverted confocal microscope (Leica Microsystems).

Reversion of EpiSC to Epi-iPS cells by stable integration of defined factors

To establish Cre-revertable transgenic EpiSC lines, 5 x 10^6 RC EpiSC were nucleofected using the Amaxa nuclefection kit (VPH-1001) with 10μg of one of the following vectors: floxed-Nanog, floxed-Esrrb, floxed-Klf4 or floxed-GFP. The transfected cells were plated in EpiSC culture conditions and stable transfectants were selected using puromycin for a period of 5 days. Following selection the cells were replated at a density of 2 x 10^5 into 2i/LIF/N2B27 in the presence of puromycin. The plates were stained for alkaline phosphatase after 7 days and scored manually. For further analysis of reverted clones, Epi-iPS cell colonies were picked and expanded in LIF/FCS/GMEMβ. Several Epi-iPS cell clones were then treated with 1μM of Tamoxifen (Sigma) for 24h to Cre-excise the transgenes, successful Cre-excision is demonstrated by the ubiquitous expression of eGFP. Efficiency of Cre-mediated transgene excision was assessed using flow cytometry. In order to test the cytokine dependency of Epi-iPS cell clones before and after Cre excision, fEsrrb and fEsrrb-Cre reverted ES cells were plated at clonal density and cultured in either LIF/FCS/GMEMβ or LIF-antagonist/FCS/GMEMβ.

PEG mediated ES x NS cell fusion experiments

RCNβH(t) NS cells were derived from the RCNβH(t) ES cell line by neural differentiation in monolayer, as described elsewhere (Pollard et al., 2006). NS cells were propagated in NS cell expansion medium in the presence of FGF and EGF (Conti et al., 2005). For all fusions:

$4x10^6$ ES cells were fused to $4x10^6$ NS cells as previously described (Silva et al., 2006) and plated in LIF/FCS/GMEMβ. For E14Tg2a, Nanog and EfEsrrb ES x E14/T NS fusions (Nanog$^{+/+}$): puromycin and G418 selections were applied one day after fusion and cell hybrids were cultured for 14 days prior to colony scoring. For Tβc44Cre6 and ESΔN-CAGE x RCNβH(t) NS fusions (Nanog$^{-/-}$): puromycin and hygromycin selection were applied one day and four days after fusion respectively. Cell hybrids were cultured for 14 days prior to colony scoring. For ESΔN-iNanog and ESΔN-iEsrrb x RCNβH(t) Red NS fusions: blasticidin and hygromycin selection were applied one day after fusion and cell hybrids were cultured for additional 15 days prior to colony scoring. Doxycycline was added only to the required plates and kept for the duration of the experiment. For ESΔN-iNanog Blue and ESΔN-iEsrrb Blue x RCNβH(t) Red NS sorted hybrids plating: 48 h after fusion cells were trypsinised and stained with 7-AAD (Molecular Probes). 7-AAD negative hybrids (live cells) were purified based on the concomitant expression of EBFP and TdTomato using a FACS-ARIA cell sorter (Becton, Dickinson). After sorting, cell purity was determined using the same FACS-ARIA cell sorter or a LSRII fortessa flow-cytometer (Becton, Dickinson) and data was analysed using the FlowJo software suite (Tree Star). Purified hybrids were replated in LIF/FCS/GMEMβ. Blasticidin and hygromycin selections were applied one day after sorting and cell hybrids were cultured for additional 15 days prior to colony scoring. Doxycycline was added to the culture medium for the duration of the experiment.

NS cells retroviral transduction for generation of pre-iPS cells

For virus preparation, 10^6 Plat-E cells per 10mm dish were seeded in FCS/GMEMβ without LIF on the day before transfection. Separate dishes of Plat-E cells were transfected with 3 μg of either pmX-Oct4, pmX-Klf4, pmX-cMyc or pmX-dsRed (Takahashi and Yamanaka, 2006)

using 27μl of Fugene6 (Promega). 24 hours after transfection the medium was replaced with 10ml of fresh GMEM/FCS and supernatant collected after additional 24 hours. Freshly harvested supernatants were filtered through a 0.45 μm cellulose acetate filter, pooled and supplemented with 4 μg/ml polybrene (Sigma). 2ml of pooled viral supernatants were added to NS cells replated at a density of 10^5 cells per 35mm dish one day before. 24 hours after infection viral supernatants were removed and NS cell were cultured for 2 additional days in NS cell expansion medium. Infected NS cells were then transferred to GMEMβ/FCS/LIF. When pre-iPS colonies started emerging, cells were replated on STO feeders in complete ES cell medium with LIF and expanded for successive experiments.

Doxycycline timecourse analysis of pre-iPS to iPS conversion

10^5 pre-iPS cells were replated on STO feeders in 35 mm dishes and cultured in LIF/FCS/GMEMβ in the presence or absence of 1 μg/ml doxycycline. After 2 days (Timecourse Day 0) cells were transferred to LIF/FCS/GMEMβ containing 1μg/ml doxycycline, 3μM 5-azacytidine, 1μg/ml doxycycline and 3μM 5-azacytidine or no drugs. Pre-iPS cells were collected everyday for 6 days for analysis using trypsin and replated in ungelatinised 10mm dishes to allow STO feeders attachment. After 1 hour unattached cells were collected and DsRed and GFP expression quantified by flow cytometry. DAPI staining was used to exclude dead cells from the analysis.

Immunoblotting

ESΔN-NERT induction: 1.3 x 10^6 ESΔN-NERT cells were plated in separate T25 flasks (IWAKI) two days before lysis. One day before lysis cell were stimulated with or without

1μM tamoxifen. For all experiments: Cell were lysed in 300 μl of a 0.5% NP-40, 50mM Tris pH 8,150mM NaCl solution with protease inhibitors (Roche). Protein extract treated with 2 μl DNA nuclease Benzonase (70664-3, Novagen) 1 h at 4°C. 50 μg of protein were denatured in Laemmli buffer at 100°C for 5 min and fractionated on a NuPage-Novex 10% Bis-Tris gels (NP0302, Invitrogen). Proteins were electro-blotted to a nitrocellulose membrane in a 25mM Tris, 0.21 M glycine, 20% methanol solution. The membrane was blocked in PBS 0.01% Tween (PBST), 10% non-fat dry milk for 2 h and incubated in 5ml of PBST 5% milk containing anti Esrrb (PP-H6707-00, Persaeus Proteomics)(1 μg/ml) or in-house made anti-Nanog rabbit polyclonal (2.4 μg /ml) antibodies or anti HDAC-2 (05-814, Upstate)(1 μg /ml) ON at 4°C or 2h at RT. Membranes were washed 3 times for 20 minutes in PBST and incubated with ECL anti-mouse IgG (NA931, Amersham) or ECL anti-rabbit IgG (NA934, Amersham) HRP conjugated secondary antibodies. Membranes were washed in PBST and developed using a Super-signal West Pico kit (Pierce) for 5 min at RT and exposed to Hyperfilm (Amersham).

Chromatin preparation

2 x 10^7 NERT ES cells were resuspended in 3ml of pre-warmed complete ES cell medium and cross-linked for 10' at RT with 1% formaldehyde (Sigma). The cross-linking reaction was stopped by adding 0.125mM glycine for 5 minutes at RT. Cells were pelleted for 3 minutes at 1300rpm at 4°C, and washed twice with cold PBS-1X (Invitrogen). Cell pellets were vigorously resuspended in 300μl of swelling buffer (5mM Pipes pH8, 85mM KCl) freshly supplemented with 1X protease inhibitor cocktail (Roche) and 0.5% NP-40. After 20 minutes on ice with occasional shaking, nuclei were spun down at 1500rpm in 15ml conical tubes (Falcon-BD) for 10 minutes at 4°C and resuspended in 1.5ml of TSE150 (0.1% SDS,

1% Triton, 2mM EDTA, 20mM Tris-HCl pH8, 150mM NaCl) buffer, freshly supplemented with 1X protease inhibitor cocktail. Samples were sonicated at 4°C in 15ml conical tubes using a Bioruptor (Diagenode) for 5 cycles of 10 minutes divided into 30 seconds ON-30 seconds OFF subcycles at maximum power. The chromatin was then transferred into 1.5ml tubes and centrifuged for 30 minutes at 14000rpm at 4°C. Soluble chromatin was divided into 250ul aliquots and stored at -80°C until use. Twenty microlitres were set apart and used to quantify the chromatin concentration and the size of the DNA fragments on a 1.5% agarose gel.

Chromatin ImmunoPrecipitation (ChIP)

For each experiment, the required amount of chromatin was thawed and pre-cleared for 1 hour and 30 minutes rotating on-wheel at 4°C in 1 ml of TSE150 containing 50µl of pA/pG sepharose beads (Sigma) 50% slurry, previously blocked with 500µg/ml of molecular grade BSA (Roche) and 1µg/ml of yeast tRNA (Invitrogen).

Pre-cleared chromatin was centrifuged at 3000rpm for 1 minute and the supernatant transferred into fresh tubes so that 20µg of DNA were used per ChIP. In addition, 20 ug of diluted chromatin was set apart for input DNA extraction and precipitation. Immunoprecipitation with in-house made anti-Nanog rabbit polyclonal (2 µg /ml) of or anti-RNAPII (Euromedex) (2 µg /ml) antibodies was performed overnight rotating on-wheel at 4°C in a final volume of 500µl of TSE150. Immunocomplexes were recovered with 50µl of blocked pA/pG sepharose beads 50% slurry for 1h and 30 minutes rotating on wheel at 4°C. Beads were recovered by centrifugation for 1 minute at 3000rpm and washed 5 minutes rotating on-wheel at RT with 1ml of buffer in the following order: TSE150, TSE500 (0.1%

SDS, 1% Triton, 2mM EDTA, 20mM Tris-HCl pH8, 500mM NaCl), washing buffer (10mM Tris-HCl pH8, 0.25M LiCl, 0.5% NP40, 0.5% Na-Deoxycholate, 1mM EDTA), and twice in TE (10mM Tris-HCl pH8, 1mM EDTA). After the last wash, elution was performed in 100µl of elution buffer (1% SDS, 10mM EDTA, 50mM Tris-HCl pH8) for 15 minutes at 65°C after vigorous vortexing. Eluates were collected after 1 minute centrifugation at 14000rpm in a benchtop microcentrifuge, and the beads rinsed in 150µl of TE-SDS1%. After 1 minute centrifugation at 14000rpm, the supernatant was pooled with the corresponding first eluate. For both immunoprecipitated and input chromatin samples, the crosslinking was reversed by incubation overnight at 65°C, followed by proteinase K treatment (Invitrogen), phenol/chlorophorm extraction and ethanol precipitation. DNA pellets were resuspended in 200µl of water. Real-time quantitative PCR reactions were performed as described. Results are presented as the average of the ChIP over input ratios normalized to enrichment detected at the Acidic Ribosomal Phosphoprotein p0 (Arbp or ArpP0) locus in three independent experiments performed on chromatin preparations from ESΔN-NERT c1, c2 and c3 ES cells.

Neural differentiation assays and laminin coating

5 x 10^4 cells were plated in 6 well plates coated overnight with Poly-L-ornithine 0.01% (P4957,Sigma), washed and coated 2 hours with laminin (CC095, Millipore) 5 µg/ml in PBS. Cells were culture in N2B27 alone (or supplemented with BMP, LIF, or BMP/LIF; made in-house) for 9 days, fixed and stained as described.

ESΔN-NERT timecourse microarray analysis

1 x 10^6 ESΔN-NERTc3 cells were plated in separate T25 flasks (IWAKI) one day before stimulation. Cells were incubated GMEMβ/FCS/LIF containing 1 μM tamoxifen for the indicated time and harvested by trypsinisation. RNA was prepared using a RNeasy minikit (Qiagen). 100 ng of RNA were reverse transcribed into ds cDNA and transcribed/amplified into biotin labelled cRNA using an Illumina TotalPrep RNA Amplification Kit (AMIL1791, Ambion). Labelled RNA was submitted to the WTCRF MRC Human Genetics Unit (University of Edinburgh) for further processing. cRNA quality was checked using a Agilent 2100 Bioanalyser and hybridization performed on an MouseWG-6 v2 BeadChip (Illumina). Raw data was processed in *R* using the *beadarray (Dunning et al., 2007)* and *limma* (Smyth, 2005) packages from the *Bioconductor* suite (Gentleman et al., 2004) Briefly, we first removed low-quality probes from the input data and then normalized sample-effects by centering individual replicate groups around the 0-hour baseline. The data was subsequently quantile-normalized and log2-transformed before assessing differential expression with the *limma* algorithms. We considered genes differentially expressed which had an FDR-adjusted p-value of at most 0.05 and a fold change of 1.5 or more for at least one time point in comparison to the 0-hour baseline.

DeepSAGE Library Preparing and Sequencing

We collected RNA and submitted the material for sample preparation and sequencing following the manufacturer's protocol for tag profiling using the *NlaIII* restriction enzyme at the GenePool core facilities of the University of Edinburgh. The RCN(t) (1 library) and RCNβH(t) (2 libraries) samples were sequenced on a first generation Illumina/Solexa

Genome Analyzer and the sorted TNG samples were sequenced at a later time point on an Illumina GAII.

Short Read Data Processing

Data analysis of short read sequencing libraries was performed using GeneProf (Halbritter et al., 2012). Briefly, reads were trimmed to the significant 17nt portion corresponding to the tag sequence and extended by the known recognition sequence of the *NlaIII* digestion enzyme (CATG). The libraries were then filtered removing low-quality reads and aligned to the Mouse reference genome (NCBIM37 assembly) using the Bowtie alignment tool (Langmead et al., 2009). Aligned reads were compared to known gene models (GeneProf reference dataset based on the Ensembl 58 database (Flicek et al., 2011)) to calculate read counts per gene, which were then quantile-normalized. We considered as differentially expressed those genes that exhibited a 1.5-fold change between groups and had a minimum (normalized) expression level of at least 10 in at least one sample.

Supplementary References

Chambers, I., Silva, J., Colby, D., Nichols, J., Nijmeijer, B., Robertson, M., Vrana, J., Jones, K., Grotewold, L., and Smith, A. (2007). Nanog safeguards pluripotency and mediates germline development. Nature *450*, 1230-1234.

Conti, L., Pollard, S.M., Gorba, T., Reitano, E., Toselli, M., Biella, G., Sun, Y., Sanzone, S., Ying, Q.L., Cattaneo, E., *et al.* (2005). Niche-independent symmetrical self-renewal of a mammalian tissue stem cell. PLoS Biol *3*, e283.

Dunning, M.J., Smith, M.L., Ritchie, M.E., and Tavare, S. (2007). beadarray: R classes and methods for Illumina bead-based data. Bioinformatics *23*, 2183-2184.

Flicek, P., Amode, M.R., Barrell, D., Beal, K., Brent, S., Chen, Y., Clapham, P., Coates, G., Fairley, S., Fitzgerald, S., *et al.* (2011). Ensembl 2011. Nucleic Acids Res *39*, D800-806.

Gentleman, R.C., Carey, V.J., Bates, D.M., Bolstad, B., Dettling, M., Dudoit, S., Ellis, B., Gautier, L., Ge, Y., Gentry, J., *et al.* (2004). Bioconductor: open software development for computational biology and bioinformatics. Genome Biol *5*, R80.

Halbritter, F., Vaidya, H.J., and Tomlinson, S.R. (2012). GeneProf: analysis of high-throughput sequencing experiments. Nat Methods *9*, 7-8.

Langmead, B., Trapnell, C., Pop, M., and Salzberg, S.L. (2009). Ultrafast and memory-efficient alignment of short DNA sequences to the human genome. Genome Biol *10*, R25.

Pollard, S.M., Conti, L., Sun, Y., Goffredo, D., and Smith, A. (2006). Adherent neural stem (NS) cells from fetal and adult forebrain. Cereb Cortex *16 Suppl 1*, i112-120.

Silva, J., Chambers, I., Pollard, S., and Smith, A. (2006). Nanog promotes transfer of pluripotency after cell fusion. Nature *441*, 997-1001.

Smyth, G.K. (2005). Limma: linear models for microarray data. In Bioinformatics and Computational Biology Solutions using R and Bioconductor, R Gentleman, V Carey, S Dudoit, R Irizarry, W Huber, eds (New York, USA: Springer Verlag), 397-420.

Takahashi, K., and Yamanaka, S. (2006). Induction of pluripotent stem cells from mouse embryonic and adult fibroblast cultures by defined factors. Cell *126*, 663-676.
